# Identification of aberrantly expressed long non-coding RNAs in stomach adenocarcinoma

**DOI:** 10.18632/oncotarget.17329

**Published:** 2017-04-21

**Authors:** Jianbin Gu, Yong Li, Liqiao Fan, Qun Zhao, Bibo Tan, Kelei Hua, Guobin Wu

**Affiliations:** ^1^ Department of General Surgery, The Fourth Affiliated Hospital of Hebei Medical University, Hebei, China

**Keywords:** stomach adenocarcinoma, expression profile, long non-coding RNA, co-expression, tumorigenesis

## Abstract

**Aim:**

Stomach adenocarcinoma (STAD) is a common malignancy worldwide. This study aimed to identify the aberrantly expressed long non-coding RNAs (lncRNAs) in STAD.

**Results:**

Total of 74 DElncRNAs and 449 DEmRNAs were identified in STAD compared with paired non-tumor tissues. The DElncRNA/DEmRNA co-expression network was constructed, which covered 519 nodes and 2993 edges. The qRT-PCR validation results of DElncRNAs were consistent with our bioinformatics analysis based on RNA-sequencing. The DEmRNAs co-expressed with DElncRNAs were significantly enriched in gastric acid secretion, complement and coagulation cascades, pancreatic secretion, cytokine-cytokine receptor interaction and Jak-STAT signaling pathway. The expression levels of the nine candidate DElncRNAs in TCGA database were compatible with our RNA-sequencing. FEZF1-AS1, HOTAIR and LINC01234 had the potential diagnosis value for STAD.

**Materials and Methods:**

The lncRNA and mRNA expression profile of 3 STAD tissues and 3 matched adjacent non-tumor tissues was obtained through high-throughput RNA-sequencing. Differentially expressed lncRNAs/mRNAs (DElncRNAs/DEmRNAs) were identified in STAD. DElncRNA/DEmRNA co-expression network construction, Gene Ontology (GO) and Kyoto Encyclopedia of Genes and Genomes (KEGG) enrichment analyses were conducted to predict the biological functions of DElncRNAs. Quantitative real-time polymerase chain reaction (qRT-PCR) was subjected to validate the expression levels of DEmRNAs and DElncRNAs. Moreover, the expression of DElncRNAs was validated through The Cancer Genome Atlas (TCGA) database. The diagnosis value of candidate DElncRNAs was accessed by receiver operating characteristic (ROC) analysis.

**Conclusions:**

Our work might provide useful information for exploring the tumorigenesis mechanism of STAD and pave the road for identification of diagnostic biomarkers in STAD.

## INTRODUCTION

Stomach cancer is one of common malignancies of digestive tract carcinoma. More than 950,000 new stomach cancer cases and 720,000 deaths occur in 2012 worldwide [1]. It is divided into stomach adenocarcinoma (STAD) and stomach squamous cell carcinoma (SSCC) based on the histopathologic feature. STAD is the predominant subtype in stomach cancer.

The regions of highest rates of stomach cancer are in Eastern Asia countries especially in China, Japan and Korea; stomach cancer is almost twice more common in males than females [1]. Numerous articles indicate that the onset and progression of stomach cancer is likely attributed to the interconnection of H. pylori infection, genetic factors and environment exposure. H. pylori is responsible for 90% new noncardia stomach cancer cases worldwide [2]. Higher expression of β2-AR, VEGF and PODXL is significantly associated with poorer survival and higher risk of recurrence in patients with stomach cancer, [3–5]. rs2494752 AG/GG variant genotype in AKT1, rs1034528 CG/CC and rs3806317 GA/GG variants in mTOR, and copy-number loss of CYCLOPS and STOP genes are related to stomach cancer susceptibility and carcinogenesis [6–8]. However, pathological mechanism of STAD is largely unknown.

microRNAs (miRNAs) with 20–25 nucleotides belong to non-coding RNAs, which function as promoting or repressing roles in cellular process tumorigenesis [9, 10]. miRNA-222 promotes cell migration by suppressing p27kip1 in STAD [11]. Over-expression of miR-296-5p promotes cell proliferation in stomach cancer by decreased expression of Caudal-related [12]. Piwi-interaction RNAs (piRNA), as a class of non-coding RNAs, is involved in tumorigenesis of several cancers. piR-823 expression is markedly reduced in gastric cancer tissues compared to non-cancerous tissues and over-expression of piR-823 suppresses cell growth in gastric cancer [13]. piR-651 is significantly up-regulated in gastric cancer and higher expression of piR-651 predicts advanced TNM stage. Knock down of piR-651 inhibits cell growth and arrests G2/M phase in gastric cancer [14]. The transcripts with longer than 200 nucleotides, not translated into proteins, are defined as long non-coding RNAs (lncRNAs) [15]. lncRNAs are known to implicate in splicing, recruiting transcription factors and mRNA stability [16]. Mounting reports demonstrate that aberrant expression of lncRNAs emerge as vital regulator of diverse biological processes in cancers, such as tumorigenesis, development and metastasis [15].

In this study, we used high-throughput RNA-sequencing to obtain lncRNA and mRNA expression profile in STAD, identified differentially expressed lncRNAs/mRNAs in STAD compared to adjacent non-tumor tissues and constructed lncRNA/mRNA co-expression network. The diagnostic value of candidate DEmRNAs was validated in TCGA database. Our study might provide valuable information for exploring tumorigenesis mechanism in STAD and identification of potential biomarkers for STAD diagnosis.

## RESULTS

### RNA-sequencing of STAD tissues

STAD and adjacent non-tumor tissues obtained from three patients were applied for RNA-sequencing. All of three patients were diagnosed as diffuse Lauren type and T4N1M0 stage of STAD (Supplementary Table 1). After raw reads trim, respective 1.05 × 10^8^, 0.95 × 10^8^, 1.15 × 10^8^ clean reads were generated from three STAD samples, and 1.04 × 10^8^, 0.89 × 10^8^, 0.968 × 10^8^ clean reads were generated from three corresponding adjacent non-tumor tissues, as Supplementary Table 2 shown. All of the clean reads were aligned with the human reference genome (hg.19). The mapped ratio of all samples was above 70% (Supplementary Table 3).

### lncRNAs and mRNAs expression profile

**Figure 1:**
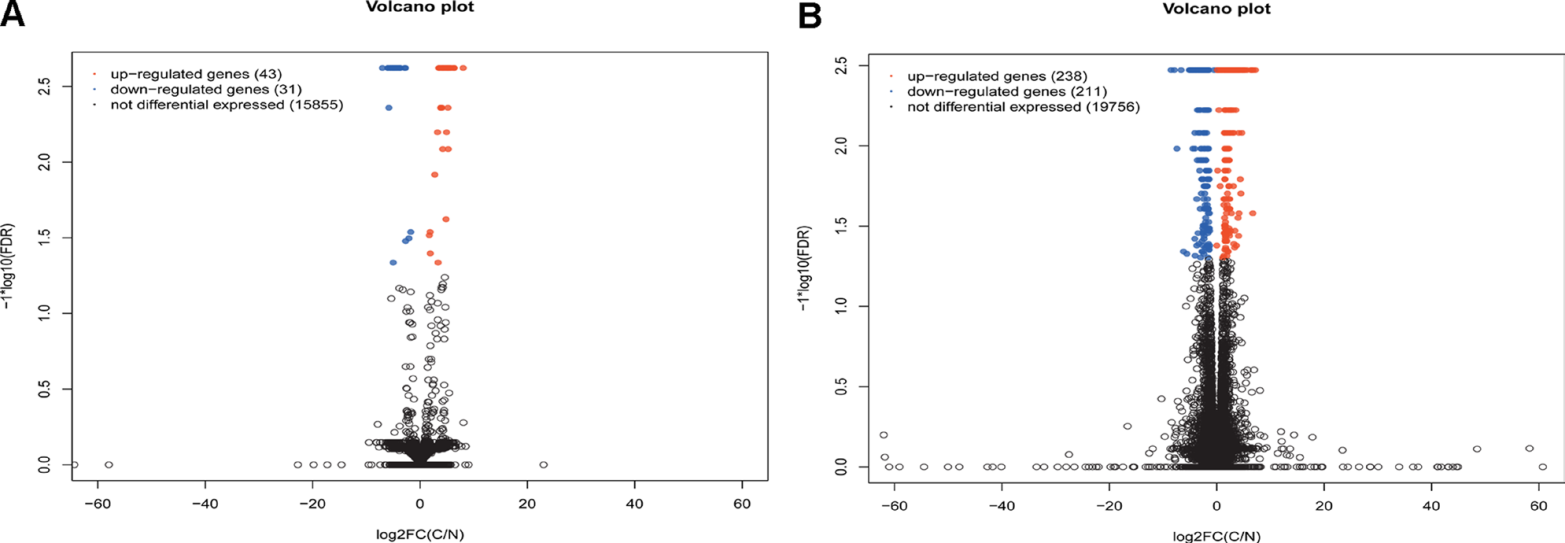
Volcano spot of lncRNA and mRNA expression in stomach adenocarcinoma (**A**) volcano spot of lncRNA expression in cancer group and control group; (**B**) volcano spot of mRNA expression in cancer group and control group. C indicated stomach adenocarcinoma and N indicated adjacent non-tumor samples. Red spot and blue spot respectively indicated up- and down-regulated genes in stomach adenocarcinoma.

Respective 15929 lncRNAs and 20205 mRNAs were mapped with the human reference genome hg. 19. Total of 74 significantly differential expression lncRNAs (DElncRNAs) with at least 2-fold change were identified in STAD compared with adjacent non-tumor tissues (Supplementary Table 4). 43 DElncRNAs were up-regulated and 31 DElncRNAs were down-regulated in STAD, which were described as volcano plot (Figure 1A). As Figure 1B shown, 449 DEmRNAs including 238 up- and 211 down-regulated DEmRNAs were screened in STAD (Supplementary Table 5).

**Figure 2:**
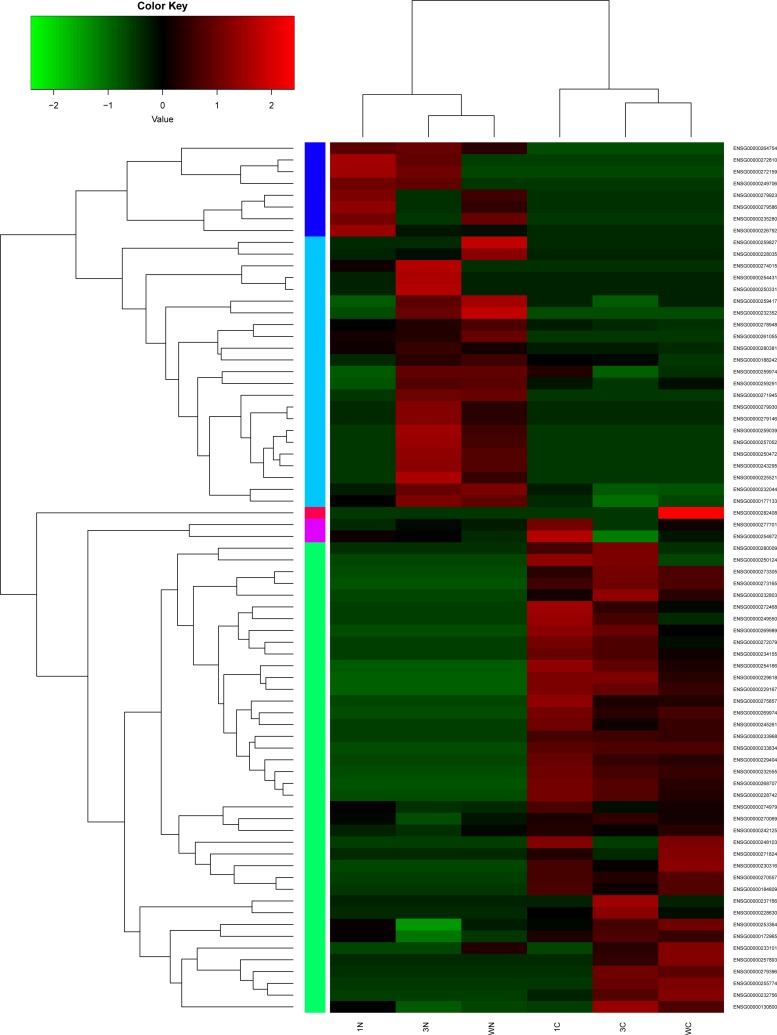
Hierarchical clustering analysis of the expression levels of DElncRNAs in stomach adenocarcinoma and adjacent non-tumor tissues Row and column represented DElncRNAs and tissue samples. The color scale indicated log_10_FPKM of the expression levels of DElncRNAs. Red and green indicated up- and down-regulation. C represented stomach adenocarcinoma tissues and N represented adjacent non-tumor tissues.

Hierarchical clustering of the lncRNAs and mRNAs expression profile was performed by using hcluster in R language. Spearman Pearson correlation between 6 samples were calculated. Hierarchical clustering of the expression of the 74 lncRNAs and 449 mRNAs displayed that there were obvious discrimination between STAD and adjacent non-tumor tissues (Figure 2 and Supplementary Figure 1). The most significantly up-regulated DElncRNA was LOC105377924 and the most significantly down-regulated DElncRNA was SEMA3B-AS1 in STAD (Table 1). S100A2 and NTRK3 were the most significantly up- and down-regulated DEmRNAs in stomach STAD, as Table 2 shown.

**Table 1:** The top 15 up- and down-regulated DElncRNAs in STAD

Ensembl Gene ID	Gene Symbol	FDR	log_2_FC
**Up-regulation**			
ENSG00000282408	LOC105377924	0.002386	8.040191
ENSG00000229618	AC011288.2	0.002386	6.394161
ENSG00000254166	CASC19	0.002386	6.274223
ENSG00000229167	RP11-73M7.1	0.002386	6.002959
ENSG00000250124	CTC-261N6.2	0.002386	5.666884
ENSG00000269989	RP11-635N19.3	0.002386	5.654653
ENSG00000272468	RP1-86C11.7	0.002386	5.428779
ENSG00000248103	CTC-338M12.9	0.002386	5.305212
ENSG00000273165	RP11-1057B6.1	0.008203	5.254069
ENSG00000230316	FEZF1-AS1	0.002386	5.249582
ENSG00000268707	RP11-247A12.7	0.004375	5.209937
ENSG00000232803	SLCO4A1-AS1	0.002386	5.176534
ENSG00000275857	AC009133.21	0.002386	5.173935
ENSG00000249550	LINC01234	0.002386	5.157594
ENSG00000269974	RP11-932O9.10	0.002386	5.095659
**Down-regulation**			
ENSG00000232352	SEMA3B-AS1	0.002386	−6.99292701
ENSG00000259417	LINC01314	0.002386	−6.006864475
ENSG00000272159	RP11-350N15.6	0.002386	−5.912151637
ENSG00000272810	U91328.22	0.004375	−5.801065509
ENSG00000259827	RP11-343H19.2	0.002386	−5.593494209
ENSG00000225521	AC005237.4	0.002386	−5.526766822
ENSG00000274015	CTD-2302E22.6	0.002386	−5.401633879
ENSG00000259039	RP11-409I10.2	0.002386	−5.179666299
ENSG00000250331	LINC01340	0.002386	−5.097315823
ENSG00000250472	TRIM36-IT1	0.002386	−5.061832349
ENSG00000264754	CTD-2653B5.1	0.002386	−5.041799308
ENSG00000232044	LINC01105	0.046115	−5.001686931
ENSG00000177133	LINC00982	0.002386	−4.981227991
ENSG00000257052	RP11-881M11.2	0.002386	−4.971847005
ENSG00000254431	RP11-550A5.2	0.002386	−4.806200304

DElncRNA: differentiated expressed long non-coding RNA; STAD: stomach adenocarcinoma; FDR: false discovery rate; FC: fold change.

**Table 2:** The top 15 up- and down-regulated DEmRNAs in STAD

Ensembl Gene ID	Gene Symbol	FDR	log_2_FC
**Up-regulation**			
ENSG00000196754	S100A2	0.00337364	7.183158953
ENSG00000186832	KRT16	0.00337364	6.789708057
ENSG00000128422	KRT17	0.0262905	6.716559208
ENSG00000241794	SPRR2A	0.00337364	6.546179879
ENSG00000170373	CST1	0.00337364	6.349039672
ENSG00000143546	S100A8	0.00337364	5.623496687
ENSG00000119547	ONECUT2	0.00337364	5.255570479
ENSG00000188100	FAM25A	0.00337364	5.221544589
ENSG00000159516	SPRR2G	0.00337364	4.984393393
ENSG00000171711	DEFB4A	0.00337364	4.874560923
ENSG00000143536	CRNN	0.00337364	4.823932742
ENSG00000125780	TGM3	0.00830821	4.667539055
ENSG00000149968	MMP3	0.00337364	4.616511381
ENSG00000163347	CLDN1	0.0198214	4.494385576
ENSG00000165474	GJB2	0.00337364	4.389336874
**Down-regulation**			
ENSG00000140538	NTRK3	0.003374	−8.49189937
ENSG00000182333	LIPF	0.003374	−7.91251678
ENSG00000157017	GHRL	0.010417	−7.41649025
ENSG00000196482	ESRRG	0.003374	−6.64138873
ENSG00000164128	NPY1R	0.045544	−6.1867236
ENSG00000168079	SCARA5	0.047017	−5.56246897
ENSG00000185615	PDIA2	0.003374	−5.084125
ENSG00000157445	CACNA2D3	0.003374	−4.95423687
ENSG00000187045	TMPRSS6	0.003374	−4.93371202
ENSG00000167779	IGFBP6	0.003374	−4.81612684
ENSG00000166165	CKB	0.003374	−4.69931608
ENSG00000196616	ADH1B	0.003374	−4.66470595
ENSG00000159197	KCNE2	0.003374	−4.53444785
ENSG00000159212	CLIC6	0.003374	−4.51924504
ENSG00000100604	CHGA	0.003374	−4.4627063

DEmRNA: differentially expressed mRNA; STAD: stomach adenocarcinoma; FDR: false discovery rate; FC: fold change.

**Figure 3:**
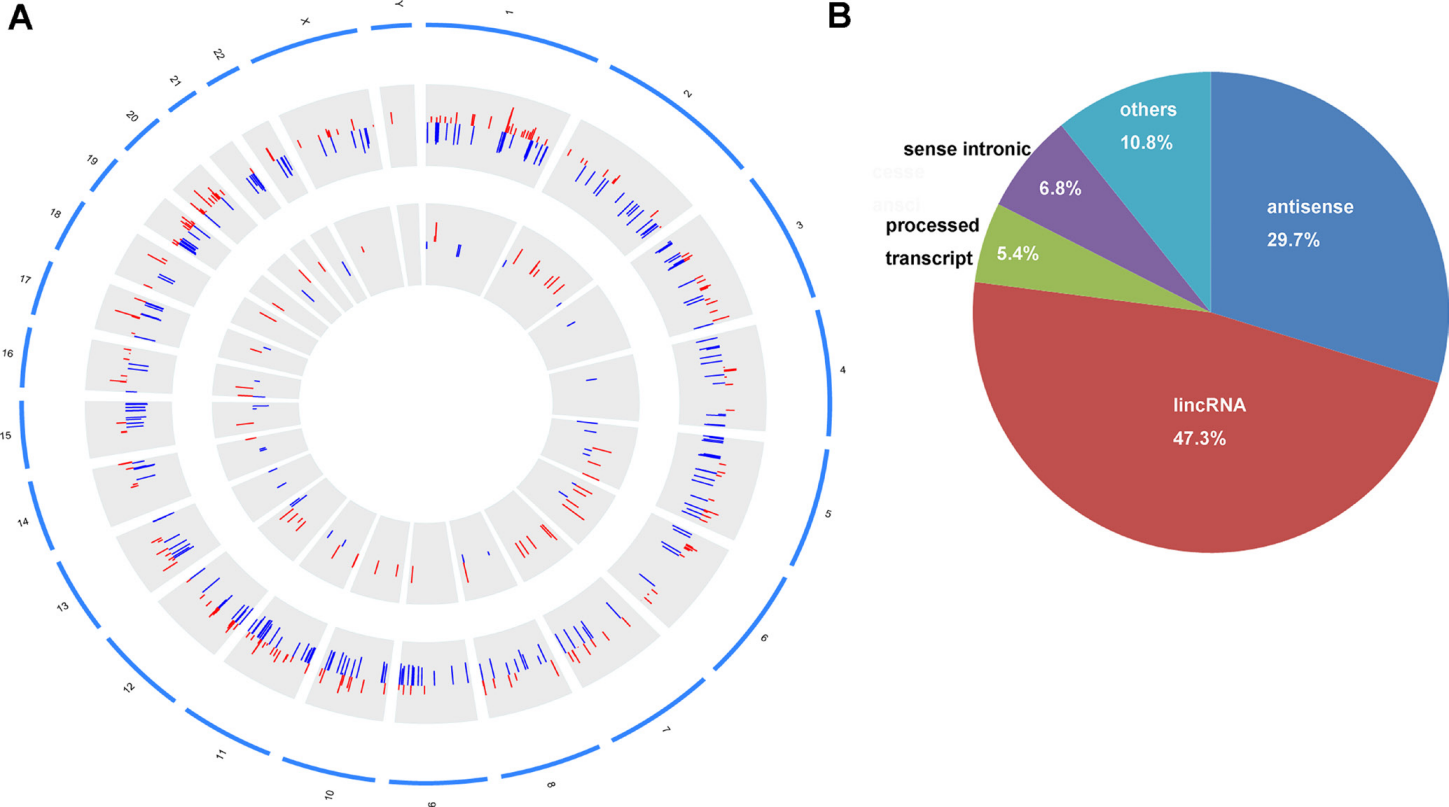
Circos plot of the distribution of DElncRNAs/DEmRNAs on chromosomes and the biotype of DElncRNAs (**A**) circos plot. The outer layer of blue cycle was the chromosome map of the human genome. The larger inner layer and smaller inner layer represented the distribution of DEmRNAs and DElncRNAs on different chromosome, respectively. Red color indicated up-regulation and blue color indicated down-regulation. The height of the bar in the larger inner layer and in the smaller inner layer represented log_2_FC of expression levels of DElncRNAs and DEmRNAs. (**B**) The biotype of 74 DElncRNAs was classified into five categories. lincRNA indicated long intergenic non-protein coding RNA.

In our study, 74 identified DElncRNAs were widely distributed in all autosomes and chromosomes X (Figure 3A). 449 DEmRNAs were distributed in all autosomes, chromosomes X and chromosomes Y (Figure 3A). Based on the annotation results, the biotype of 74 DElncRNAs was divided into 5 categories. 22 DElncRNAs (29.7%) were natural antisense, 35 DElncRNAs (47.3%) were lincRNAs, 4 DElncRNAs (5.4%) were processed transcripts, 5 DElncRNAs (6.8%) was sense intronic and 8 DElncRNAs (10.8%) were others (Figure 3B).

### DElncRNA/DEmRNA co-expression network

**Figure 4:**
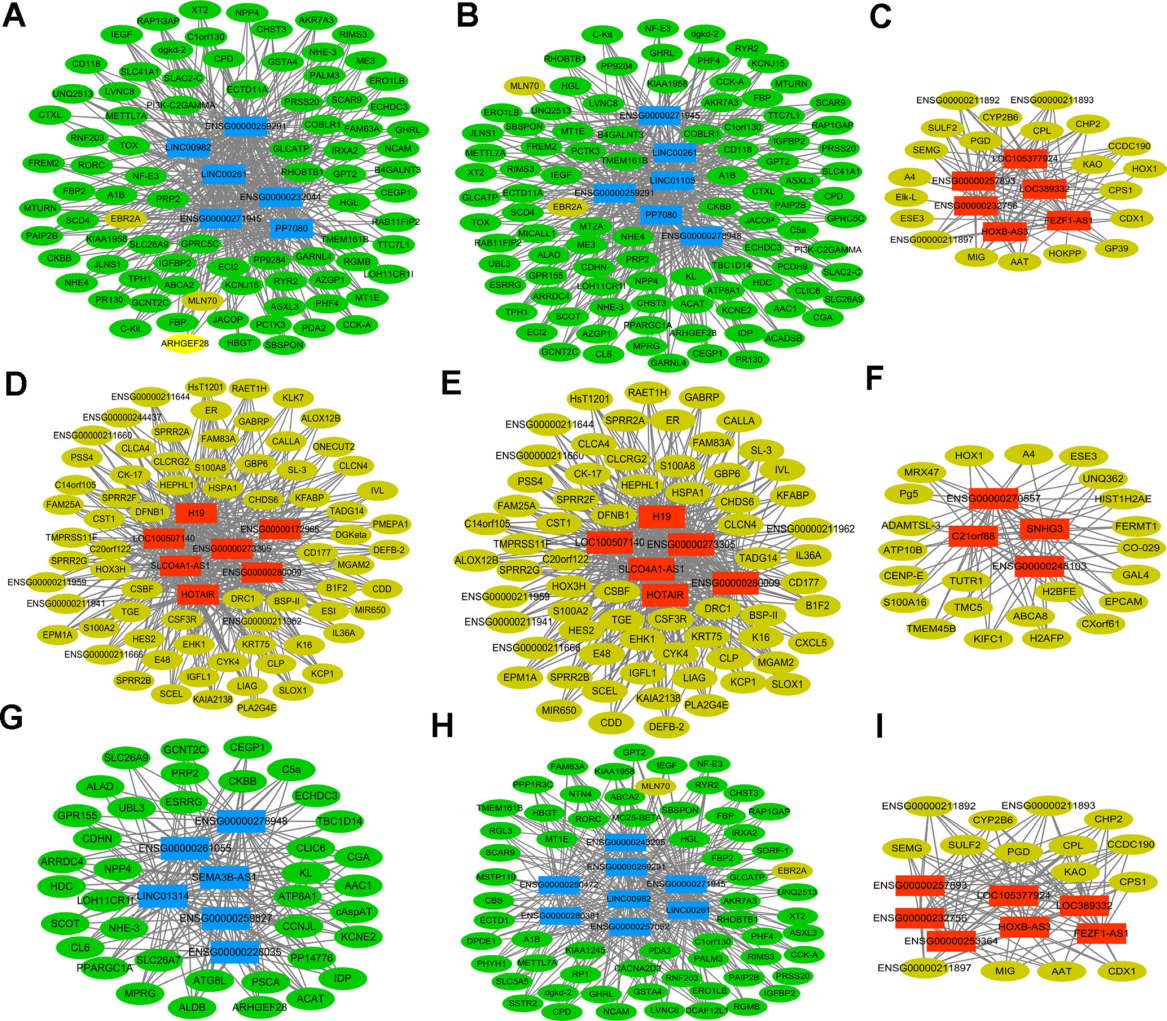
Sub-networks of DElncRNAs/DEmRNAs co-expression (**A**) the sub-network of LINC00261; (**B**) the sub-network of LINC01105; (**C**) the sub-network of FEZF1-AS1; (**D**) the sub-network of H19; (**E**) the sub-network of HOTAIR; (**F**) the sub-network of SNHG3; (**G**) the sub-network of SEMA3B-AS1; (**H**) the sub-network of LINC00982; (**I**) the sub-network of LINC105377924. Circular node represented DEmRNA and rectangle node represented DElncRNA. Yellow was up-regulated DEmRNA and green was down-regulated DEmRNAs; Red indicated up-regulated DElncRNA and blue indicated down-regulated DElncRNA.

In order to investigate the potential functions of DElncRNAs in STAD, the Pearson correlation coefficient (PCC) indicating the co-expression relationship between 74 DElncRNAs and 449 DEmRNAs were calculated based on the expression levels of DElncRNAs and DEmRNAs. DElncRNA/DEmRNA co-expression pairs with |PCC| ≥ 0.9 were our concerns. In Supplementary Figure 2, total of 74 DElncRNAs and 445 DEmRNAs were involved in the co-expressed network, which consisted of 519 nodes and 2993 edges. LINC01105 (ENSG00000232044, Figure 4B), RP11-440D17.4 (ENSG00000273305), RP11-617F23.1 (ENSG00000259291) and LINC00261 (ENSG0000 0259974, Figure 4A) were the hub DElncRNAs in the network, which had high connectivity with DEmRNAs.

### GO and KEGG pathway enrichment analyses

In order to predict the potential functions of DEmRNAs co-expressed with DElncRNAs, GO and KEGG pathway were adopted to demonstrate it. 445 DEmRNAs co-expressed with DElncRNAs were significantly enriched in extracellular matrix and extracellular region of cell component, cell adhesion and biological adhesion in biological process, calcium ion binding and receptor binding in molecular function (Table 3). Those DEmRNAs were significantly enriched in several KEGG signaling pathways. Gastric acid secretion, complement and coagulation cascades, pancreatic secretion, cytokine-cytokine receptor interaction and Jak-STAT signaling pathway were the top pathways associated with STAD (Table 4).

**Table 3:** GO term enrichment of dysregulated mRNAs

GO ID	GO terms	*P*-value	FDR
**Cell component (top 10)**			
GO:0031012	extracellular matrix	8.79E-11	4.84E-07
GO:0005576	extracellular region	1.19E-10	6.57E-07
GO:0031988	membrane-bounded vesicle	1.60E-10	8.79E-07
GO:0005615	extracellular space	1.64E-10	9.05E-07
GO:0031982	vesicle	2.14E-10	1.18E-06
GO:0044421	extracellular region part	2.22E-10	1.22E-06
GO:0070062	extracellular vesicular exosome	2.78E-10	1.53E-06
GO:0043230	extracellular organelle	2.80E-10	1.54E-06
GO:0065010	extracellular membrane-bounded organelle	2.80E-10	1.54E-06
GO:0005578	proteinaceous extracellular matrix	6.97E-09	3.84E-05
**Biological process (top 10)**			
GO:0007155	cell adhesion	1.83E-10	1.01E-06
GO:0022610	biological adhesion	2.16E-10	1.19E-06
GO:0044763	single-organism cellular process	2.75E-10	1.52E-06
GO:0044699	single-organism process	3.15E-10	1.73E-06
GO:0050896	response to stimulus	2.85E-09	1.57E-05
GO:0006950	response to stress	6.20E-09	3.42E-05
GO:0032101	regulation of response to external stimulus	7.94E-09	4.37E-05
GO:0030198	extracellular matrix organization	8.31E-09	4.58E-05
GO:0043062	extracellular structure organization	8.84E-09	4.87E-05
GO:0009611	response to wounding	1.42E-08	7.85E-05
**Molecular function (top 10)**			
GO:0005509	calcium ion binding	2.63E-08	0.000145
GO:0005102	receptor binding	1.16E-07	0.000636
GO:0004866	endopeptidase inhibitor activity	5.07E-06	0.0279
GO:0061135	endopeptidase regulator activity	7.81E-06	0.043
GO:0005254	chloride channel activity	8.67E-06	0.0478
GO:0030414	peptidase inhibitor activity	8.97E-06	0.0494

FDR: false discovery rate.

**Table 4:** KEGG pathway enrichment of dysregulated mRNAs in STAD

Items	Items_Details	FDR	Genes
hsa04971	Gastric acid secretion	1.98E-05	SLC9A4, CALML3, CFTR, ADCY2, KCNE2, KCNJ15, SLC26A7, KCNQ1, SSTR2
hsa04610	Complement and coagulation cascades	5.21E-05	F10, F2R, SERPINA1, C5, C8A, F5, CR2, C7
hsa04972	Pancreatic secretion	9.20E-05	CFTR, CLCA4, ADCY2, CLCA2, KCNQ1, RYR2, TRPC1, CCKAR, PLA2G4E
hsa04640	Hematopoietic cell lineage	0.001457	KIT, ITGA2, CSF3R, CSF3, CR2, MME, IL11RA
hsa05322	Systemic lupus erythematosus	0.001691	HIST1H2BM, C5, C8A, HIST1H2AE, HIST1H2BE, FCGR3A, C7
hsa05020	Prion diseases	0.001992	C5, C8A, C7
hsa00480	Glutathione metabolism	0.002214	IDH2, GSTP1, RRM2, PGD, GSTA4
hsa00980	Metabolism of xenobiotics by cytochrome P450	0.002473	AKR1C4, CYP2B6, DHDH, GSTP1, GSTA4, ADH1B
hsa04060	Cytokine-cytokine receptor interaction	0.002843	KIT, CXCL9, CXCL5, CXCL1, CCR8, LIFR, EDA, TNFSF15, CSF3R, CSF3, IL11RA
hsa00590	Arachidonic acid metabolism	0.002877	CYP2B6, ALOX12B, PTGIS, PLA2G4E, PTGS2
hsa04640	Hematopoietic cell lineage	0.002996	KIT, CSF3R, CSF3, IL11RA
hsa00010	Glycolysis / Gluconeogenesis	0.00308	FBP2, ALDOB, HKDC1
hsa00051	Fructose and mannose metabolism	0.00308	FBP2, ALDOB, HKDC1
hsa04970	Salivary secretion	0.003109	CALML3, ADCY2, MUC5B, DMBT1, AQP5, CST1
hsa04978	Mineral absorption	0.003124	MT2A, SLC26A9, HEPH, MT1E, SLC9A3
hsa04020	Calcium signaling pathway	0.003165	ADCY2, RYR2, TRPC1, CCKAR
hsa05200	Pathways in cancer	0.003449	HIF1A, KIT, LAMA2, ITGA2, CSF3R, FGF13, GSTP1, WNT5A, BRCA2, LAMC2, PTGS2, MMP1
hsa05412	Arrhythmogenic right ventricular cardiomyopathy	0.007626	RYR2, LAMA2, ITGA2, CACNA2D3, CDH2
hsa04630	Jak-STAT signaling pathway	0.007986	CSF3R, CSF3, IL11RA
hsa00532	Glycosaminoglycan biosynthesis - chondroitin sulfate	0.00833	XYLT2, B3GAT1, CHST3

STAD: stomach adenocarcinoma; FDR: false discovery rate.

Moreover, those DEmRNAs (underlined genes in Table 4) co-expressed with DElncRNAs shown in Figure 3 were significantly enriched in abovementioned signaling pathways. KCNE2 (co-expressed with LINC01105 and SEM3B-AS1), KCNJ15 (co-expressed with LINC01105 and LINC00261), SLC26A7 (co-expressed with SEM3B-AS1) and SSTR2 (co-expressed with LINC00982) were significantly enriched in gastric acid secretion; CLCA4 co-expressed with H19 and HOTAIR was significantly enriched in pancreatic secretion; CSF3R co-expressed with H19 and HOTAIR was significantly enriched in pathways in cancer, Jak-STAT signaling pathway and cytokine-cytokine receptor interaction; CXCL5 co-expressed with H19 and HOTAIR was significantly enriched in cytokine-cytokine receptor interaction; RYR2 co-expressed with LINC00261 and LINC01105 was significantly enriched in pancreatic secretion and calcium signaling pathway.

### qRT-PCR validation of DElncRNA and DEmRNAs

**Figure 5:**
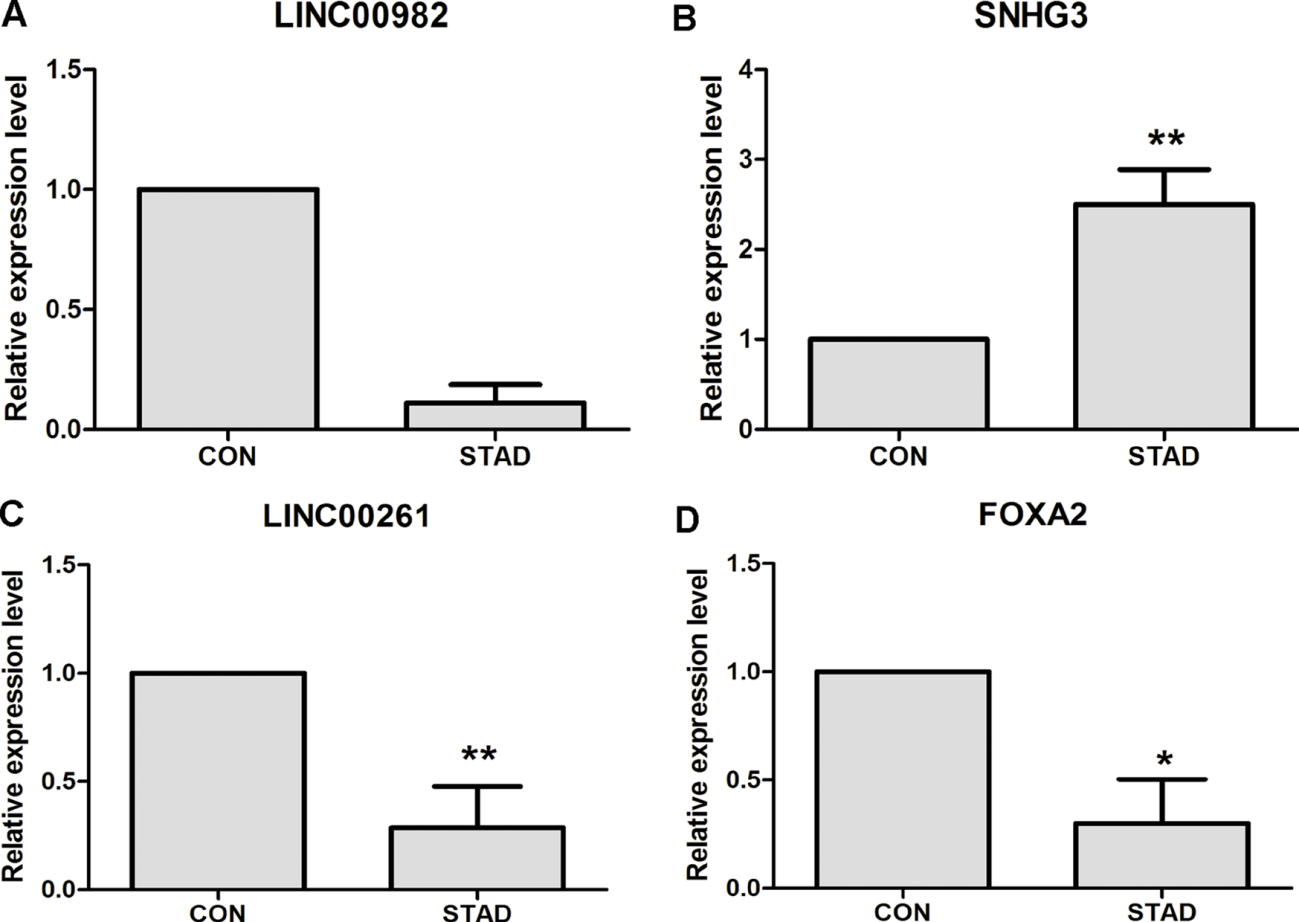
qRT-PCR validation of dysregulated DElncRNAs and DEmRNAs in STAD compared with matched non-tumor tissues (**A**) The expression level of lncRNA LINC00982; (**B**) The expression level of lncRNA SNHG3; (**C**) The expression level of lncRNA LINC00261; (**D**) The expression level of FOXA2. STAD indicated stomach adenocarcinoma; CON indicated paired adjacent non-tumor tissues. * represented *P* < 0.05 and ** represented *P* < 0.01.

DElncRNAs and DEmRNAs were identified in our study through high throughout sequencing and bioinformatics analysis. qRT-PCR was subjected to validate the expression levels of DElncRNAs and DEmRNAs in ten STAD tissues and ten matched adjacent tumor tissues. Three DElncRNAs and one DEmRNAs were chose for verification. As Figure 5A and 5C shown, LINC00982 and LINC00261 (*P* < 0.01) were down-regulated in STAD compared with controls. SHNG3 was significantly up-regulated in STAD tissues in Figure 5B (*P* < 0.01). In our DEmRNA expression profile analysis, FOXA2 was not significantly down-regulated (FDR > 0.05), but it had the down-regulated tendency in STAD. In the qRT-PCR validation, FOXA2 (*P* < 0.01) was significantly down-regulated in STAD (Figure 5D). In generally, the verification results of qRT-PCR were consistent with our bioinformatics analysis.

### The expression levels of candidate DElncRNAs were analyzed in the TCGA datasets

**Figure 6:**
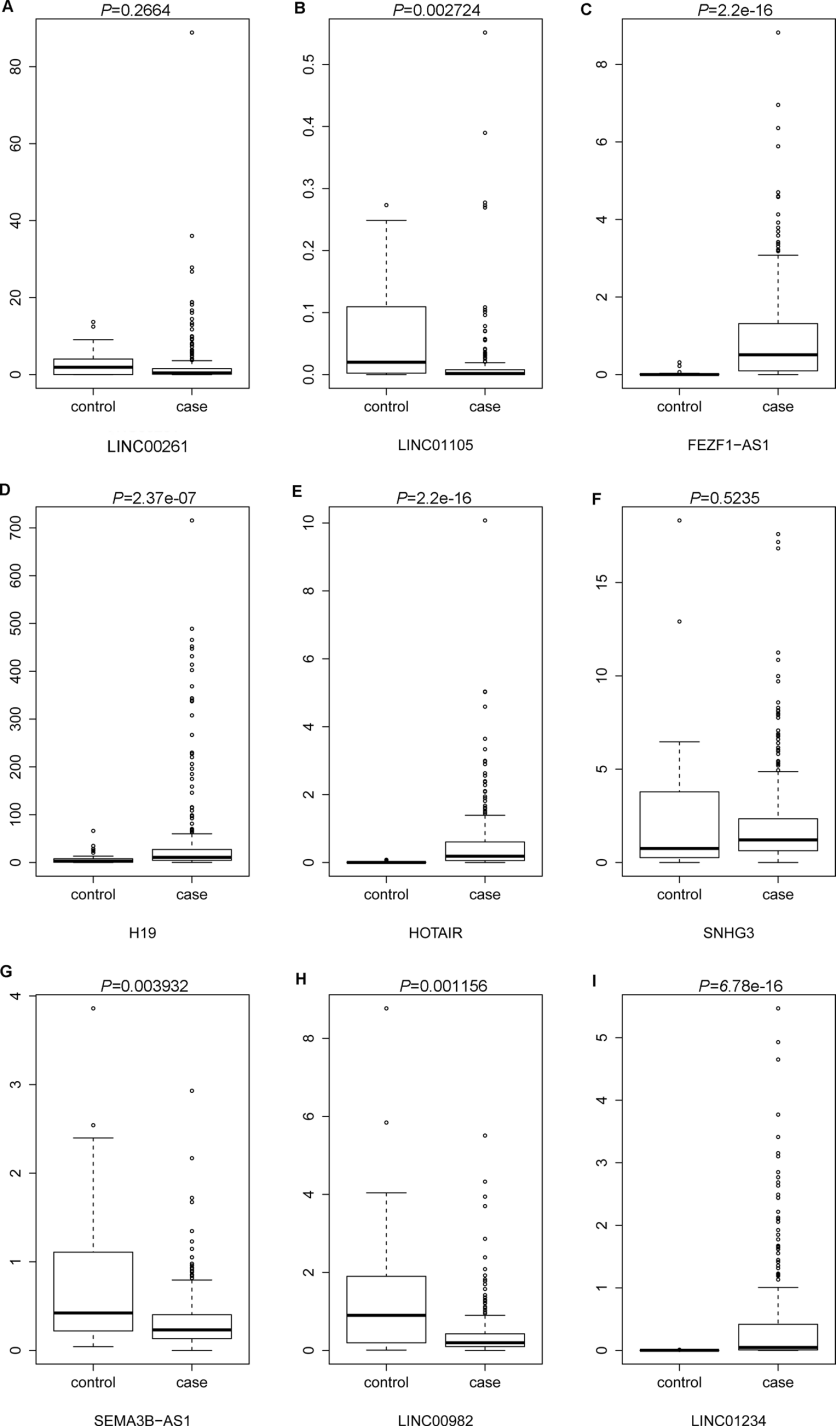
The cross validation of the expression levels of candidate DElncRNAs in STAD tissues based on TCGA database (**A**) LINC00261; (**B**) LINC01105; (**C**) FEZF1-AS1; (**D**) H19; (**E**) HOTAIR; (**F**) SNHG3; (**G**) SEMA3B-AS1; (**H**) LINC00982; (**I**) LINC01234. Case group and control group indicated STAD tissues and adjacent non-tumor tissues.

The lncRNA expression profile of 285 STAD tissues (case group) and 33 adjacent non-tumor tissues (control group) were retrieved from TCGA database. The difference of expression levels of the nine DElncRNAs between STAD and non-tumor tissues were analyzed and were depicted using box-plots, which was visually illustrated by median and inter-quartile range. As Figure 6B, 6G and 6H shown, the expression levels of LINC01105 (*P* = 0.002724), SEMA3B-AS1 (*P* = 0.003923) and LINC00982 (*P* = 0.001156) were significantly down-regulated in case group compared with control group. In addition, the expression levels of FEZF1-AS1 (*P* = 2.2e-16), H19 (*P* = 2.37e-07), HOTAIR (*P* = 2.2e-16) and LINC01234 (*P* = 6.78e-16) in case group were significantly higher than that in control group, as Figure 6C–6E and 6I revealed. In addition, the difference of expression levels of LINC00261 and SNHG3 between case group and control group was not significant, but LINC00261 expression had the down-regulated tendency and SNHG3 expression had the up-tendency in case group compared with control group (Figure 6A and 6F). In general, the results of cross validation indicated that the expression patterns of candidate DElncRNAs in TCGA database was compatible with our RNA-sequencing and bioinformatics analyses.

### ROC curve analysis

**Figure 7:**
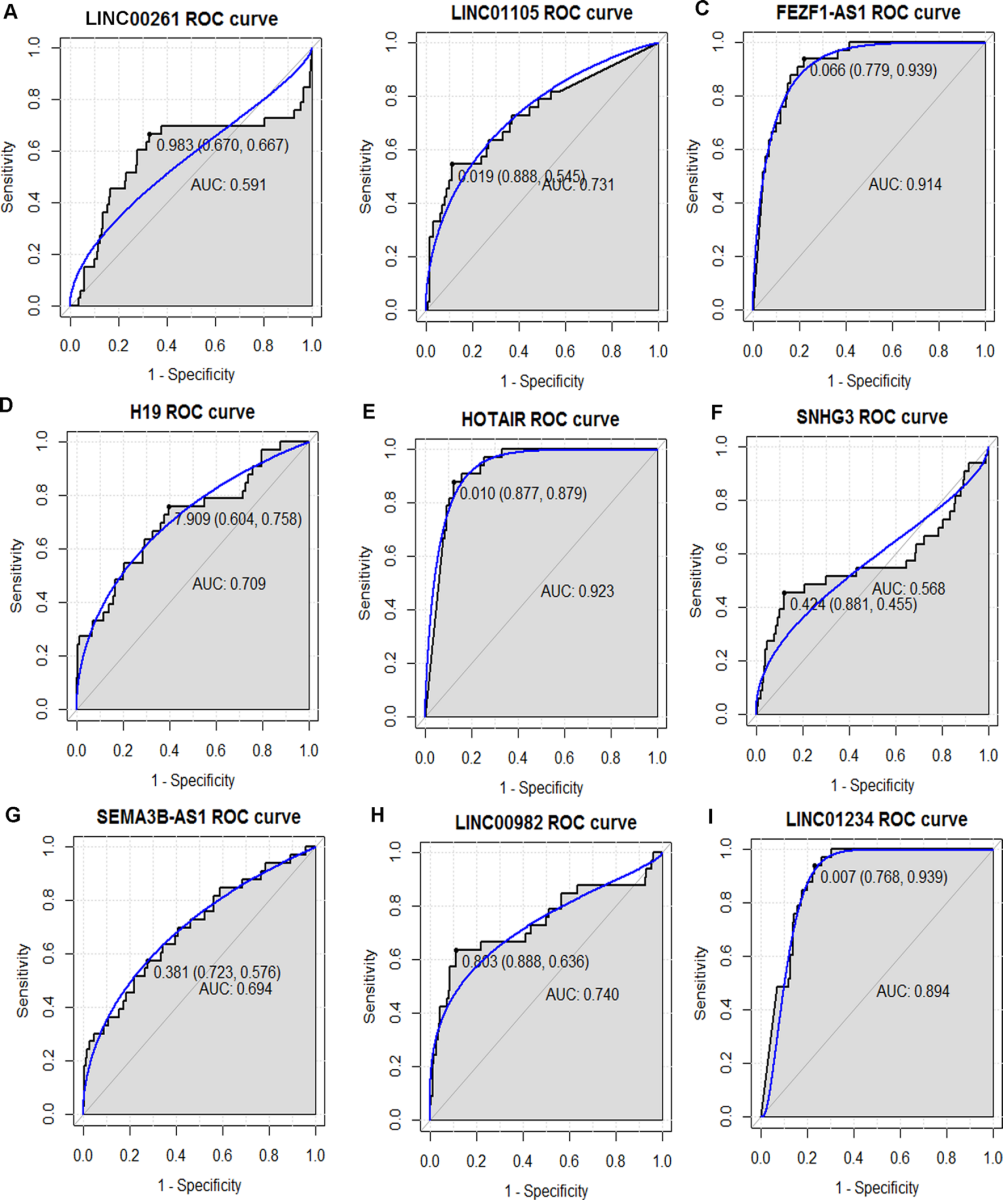
The discriminatory ability of the evaluated DElncRNAs between STAD tissues and adjacent non-tumor tissues was accessed with ROC curve (**A**) LINC00261; (**B**) LINC01105; (**C**) FEZF1-AS1; (**D**) H19; (**E**) HOTAIR; (**F**) SNHG3; (**G**) SEMA3B-AS1; (**H**) LINC00982; (**I**) LINC01234.

In order to access the discriminatory ability of the nine candidate DElncRNAs among 285 STAD tissues and 33 adjacent non-tumor tissues generated from TCGA database, ROC curve analyses were conducted and AUC were calculated. As Figure 7B–7E, 7H and 7I shown, the AUC of LINC01105 (0.731), FEZF1-AS1 (0.914), H19 (0.709), HOTAIR (0.923), LINC00982 (0.740) and LINC01234 (0.894) was more than 0.7; the AUC of LINC00261 (0.591, Figure 7A), SNHG3 (0.568, Figure 7F) and SEMA3B-AS1 (0.694, Figure 7G) was less than 0.7.

FEZF1-AS1, HOTAIR and LINC01234 had the largest AUC in those nine DElncRNAs. For STAD diagnosis, the sensitivity and specificity of FEZF1-AS1 was 93.9% and 77.9% (Figure 7C); the sensitivity and specificity of HOTAIR was 87.9% and 87.7% (Figure 7E); the sensitivity and specificity of LINC01234 was 93.9% and 76.8% (Figure 7I), respectively.

## DISCUSSION

LncRNAs are long non-coding transcripts with longer than 200 nucleotides. They are expressed at lower level compared to protein-coding mRNA [17]. Mounting evidence demonstrate that DElncRNAs are linked to multiple human diseases, such as colorectal cancer, breast cancer and lung cancer [18].

In our study, the landscape of lncRNAs in STAD tissues was obtained by RNA-sequencing . Total of 74 DElncRNAs and 449 DEmRNAs were identified in STAD. The expression levels of LINC00982, LINC00261 and SNHG3 of DElncRNAs and FOXA2 of DEmRNA were verified through qRT-PCR, the results of which were generally consistent with high-throughput sequencing. DElncRNAs distributed on all chromosomes except Y chromosomes and DEmRNAs distributed on all chromosomes including X and Y chromosomes. The DElncRNA/DEmRNA co-expression network was constructed for predicting the potential functions of DElncRNAs in STAD.

In accordance with the previous studies, lncRNA H19 and lncRNA HOTAIR were up-regulated and LINC00261 was down-regulated in stomach cancer. In our data, H19 and HOTAIR was up-regulated with 23.5 and 17.5 fold in STAD, respectively. H19 co-expressed with 72 DEmRNAs and 6 DElncRNAs (Figure 4D). Increased expression of H19 contributes to tumor suppressor p53 inactivation in stomach cancer, which promotes cell proliferation, migration, invasion and metastasis in stomach cancer [19, 20]. Increased H19 is significantly correlated to advanced TNM stage and lymph node metastasis, while leads to poorer overall survival in patients with STAD [21]. Circulating H19 is up-regulated in plasma of patients with STAD compared to healthy controls and it might be promising biomarker of STAD patients [22]. In the DElncRNA/DEmRNA co-expression network, HOTAIR positively correlated with 67 DEmRNAs and 5 DElncRNAs, such as H19 (Figure 4E). Over-expression of HOTAIR promotes the epithelia-to-mesenchymal transition, cell invasion, cell metastasis and it is associated with unfavorable survival of patients with STAD [23–26].

LINC00982 and LINC00261 were down-regulated and SNHG3 significantly up-regulated in SATD (Figure 3), which were consistent with our high-throughput sequencing. The recent study demonstrates that decreased LINC000982 contributes to cell proliferation, cell cycle progression, poor overall survival and disease-free survival; and its over-expression suppresses cell proliferation and renders cell cycle arrest in stomach cancer [27]. LINC00261 is down-regulated in stomach cancer and co-expressed with 85 DEmRNAs in our study (Figure 4A). Lower expression of LINC00261 correlates with deeper tumor invasion, poorer differentiation and lymph node metastasis in stomach cancer [28]. Currently, the potential functions and mechanism of SNHG3 in STAD are largely unclear. Our study was the first time to report that SNHG3 was over-expressed in STAD. It is reported that SNHG3 is significantly up-regulated in hepatocellular carcinoma (HCC) compared with paired non-tumor tissues; up-regulation of SNHG3 is correlated with overall survival, recurrence-free survival and relapse in HCC [29]. The aforementioned information suggests that increased SNHG3 might contribute to the carcinogenesis of STAD.

FEZF1-AS1 is up-regulated in colorectal carcinoma (CRC), which promotes aggressive behaviors of CRC cells, including cell proliferation, cell migration and cell metastasis [30]. In our results, FEZF1-AS1 was one of top 10 significantly up-regulated DElncRNAs with 38 fold up-regulation in STAD. FEZF1-AS1 was identified as a novel lncRNA associated with STAD tumorigenesis in our study. LINC01234 is up-regulated in esophageal squamous cell carcinoma (ESCC) and the three-lncRNA signature including LINC01234 could accurately predict survival of patients with ESCC [31, 32]. LINC01234 had almost 36 fold up-regulation in STAD. The potential roles of LINC01234 in STAD tumorigenesis need to be explored in the future work.

445 out of 449 DEmRNAs were co-expressed with 74 DElncRNAs. In order to predict the potential functions of DElncRNAs, GO terms and KEGG signaling pathways of 445 DEmRNAs were enriched, respectively. The results of significant GO annotation, such as cell adhesion, biological adhesion, calcium ion binding and receptor binding, indicated that DElncRNAs and DEmRNAs associated with these GO terms might contribute to STAD tumorigenesis. Several KEGG pathways were enriched from DEmRNAs, such as gastric acid secretion, complement and coagulation cascades, pancreatic secretion, cytokine-cytokine receptor interaction and Jak-STAT signaling pathway, suggesting that DElncRNAs and DEmRNAs might play essential roles through involving in those pathways in STAD.

Except of H19, HOTAIR, LINC000982 and LINC00261, most of DElncRNAs were identified as novel lncRNAs associated with STAD, such as the most significantly up-regulated LOC105377924 (Figure 4I), the most significantly down-regulated SEMA3B-AS1 (Figure 4G), and LINC01105 (ENSG00000232044, Figure 4B), which had the highest degree with DEmRNAs in the co-expression network. Whether those novel DElncRNAs refer to the initiation and development of STAD needs to be clarified through *in vivo* and *in vitro* experiments in the future work. The ROC curve analyses indicated that lncRNA FEZF1-AS1, HOTAIR and LINC01234 might be potential biomarkers for STAD diagnosis. In the further work, large cohort of STAD patients needs to be enrolled and the discriminatory ability of those DElncRNAs in STAD diagnosis needs to be further validated in clinical practice.

Our work reported the dysregulated lncRNAs involved in STAD by using high-throughput RNA sequencing. Our study might be the foundation for future investigation for diagnostic biomarkers for STAD and illumination of tumorigenesis mechanisms of STAD.

## MATERIALS AND METHODS

### Patients and samples

STAD tissues and adjacent non-tumor tissues were obtained from patients received the tumor radical resection in the Fourth Affiliated Hospital of Hebei Medical University. Patients were received none of chemo- or radiotherapy before gastrectomy. The pathological type of patients with stomach cancer was explicitly diagnosed by histopathologic examination. The details of clinical features including gender, age, Lauren type, histologic type and TNM stage were collected. Lastly, 3 subjects with STAD were enrolled into our study.

The study was approved by the Ethics Committee of the Fourth Affiliated Hospital of Hebei Medical University and informed written consent was obtained from all patients. The research complied with the principles of the Declaration of Helsinki.

### Library preparation and high-throughput sequencing

Total RNA of collected specimens was extracted by TRIzol reagent (Invitrogen, Carlsbad, CA, USA) according to the manual instruction. Firstly, total RNA was subjected to ribosomal RNA removal by Ribo-Zero Magnetic kit (EpiCentre, Mandison, WI, USA); secondly, cDNA library was constructed by Truseq RNA sample Prep Kit (Illumina, Inc., San Diego, CA, USA). RNA was purified and fragmented into 200 base pairs; RNA fragment primed with random hexamer primers were applied for the first cDNA strand synthesis; then the second cDNA strand was synthesized and dUTP was used instead of dTTP; the double cDNA strands were underwent end pair, 3′end adenylation and adapter ligation; the second cDNA strand was digested by UNG enzyme (Illumina, Inc., San Diego, CA, USA). Thirdly, libraries were amplified through polymerase chain reaction for 15 cycles, purified through Certified Low Range Ultra Agarose (Bio-Rad) and quantified through Picogreen (Molecular probes) on TBS380 (Turner Biosystems). Then bridge PCR was performed on cBot. Lastly, each library was loaded into one lane of the Illumina HiSeq 4000 for sequencing.

### Quality control of raw sequence

The raw image data obtained from high-throughput RNA-sequencing was translated into raw FASTQ sequence data by Base Calling. Raw RNA-Seq data were filtered using FASTxtool SeqPrep (https://github.com/jstjohn/SeqPrep) and Sickle (https://github.com/najoshi/sickle) according to the following criteria: (1) reads containing sequencing adaptors; (2) nucleotides with a quality score < 20 were trimmed from the end of the sequence; once the quality value of reads after being trimmed less than 10, the whole sequence was abandoned; (3) N base rate of raw reads with more than 10% were discarded; (4) raw reads with base pairs less than 20 after adapter removal and trim was abnegated.

### Clean reads mapping

Clean reads were aligned with the human reference genome, Ensemble GRCh38 v 84 (hg19), by using TopHat and Ensemble gene annotation. TopHat allows a maximum of two mismatches when mapping the reads to the reference genome. Fragment assembly and the relative expression of the reads with the normalized RNA-Seq fragment counts were processed by Cufflinks [33]. The assembled transcripts were merged to an integrated transcriptome of by using Cuffmerge. Fragments per kilobase of exon per million mapped reads (FPKM) were used to determine the transcription abundance of lncRNA and mRNA.

### Differentially expressed lncRNA and differentially expressed mRNA analysis

Cuffdiff was used to calculate the FPKM of each reads. Paired *t*-tests were carried out for expression testing between STAD and adjacent non-tumor tissues [33]. FDR < 0.05 and |log_2_FC > 1| was selected as the criteria for DElncRNAs and FDR < 0.05 was set as the threshold of DEmRNAs after applying Benjamini-Hochberg correction for multiple test.

### Co-expression analysis and network construction between DElncRNAs and DEmRNAs

DElncRNA-DEmRNA co-expression network was performed to explore the critical roles of DElncRNA and DEmRNA in progression of STAD. Pearson correlation coefficient (PCC) reflecting co-expression relationship between DElncRNAs and DEmRNAs were calculated according to their expression levels. DElncRNA-DEmRNA pairs with |PCC| ≥ 0.90 were retained for network construction [34]. The DElncRNA-DEmRNA network was visualized by Cytoscape 3.1 (http://cytoscape.org/) [35].

### Gene Ontology and KEGG enrichment analysis

Gene Ontology (GO) function and Kyoto Encyclopedia of Genes and Genomes (KEGG) pathway enrichment analyses were used to predict the biological function of DEmRNAs [36]. Online GeneCoDis3 (http://genecodis.cnb.csic.es/analysis) was used to enrich GO terms and KEGG pathways [37, 38]. FDR < 0.05 was set as the cut-off for significant GO terms and KEGG pathways.

### Quantitative real-time polymerase chain reaction (qRT-PCR)

Total RNA of STAD and adjacent non-tumor tissues were extracted using Trizol (Invitrogen, CA, USA) according to the manual instructions. The M-MLV Reverse Transcriptase kit (Promega, WI, USA) was used to synthesize the cDNA. qRT-PCR reactions were performed using SYBR Green PCR Master Mix (Applied Biosystems, Foster City, CA) on the Rotor gene 3000 (Corbett, Brisbane, Australia). β-actin was used as internal control for mRNA detection. The relative expression of candidate genes was calculated using the 2^−ΔΔCT^ equation [39]. The PCR primers were shown as Supplementary Table 6. At least triple experiments were subjected to qRT-PCR verification.

### The expression levels of DElncRNAs were validated through lncRNA expression profile generated from The Cancer Genome Atlas database

The Cancer Genome Atlas (TCGA, https://tcga-data.nci.nih.gov/tcga/) is a public funded project and has produced multidimensional data at the DNA, RNA and protein levels for a broad range of human tumor types. The Atlas of Noncoding RNAs in Cancer (TANRIC, http://ibl.mdanderson.org/tanric/_design/basic/index.html) is an open resource for interactive exploration of lncRNAs in the context of TCGA clinical and genomic data. In our study, TANRIC database was employed to retrieve the lncRNA expression profile of STAD based on RNA-sequencing generated from TCGA data. Lastly, the lncRNA expression data of 285 STAD tissues and 33 adjacent non-tumor tissues were retrieved. The difference of expression levels of DElncRNAs between STAD tissues and adjacent non-tumor tissues were compared and were delineated by box-plot.

### Receiver operating characteristic analyses

In order to assess the diagnostic value of candidate DElncRNAs, receiver operating characteristic (ROC) analyses were performed using pROC package in R language. The area under the curve (AUC) under binomial exact confidence interval was calculated to generate the ROC curve.

### Statistical analysis

Mean ± standard deviation and independent-samples *t*-test were used in the statistical analysis. *P* < 0.05 was considered as significant difference. To group samples based on expression values of mRNA and lncRNAs, we performed hierarchical cluster analysis [40]. These analyses were performed by R platform (http://www.r-project.org/) [41]. Circos plot was described as the distribution of DElncRNAs and DEmRNAs in chromosomes.

## SUPPLEMENTARY MATERIALS FIGURES AND TABLES






