# Supplementary material for: Identification of aberrantly expressed long non-coding RNAs in stomach adenocarcinoma
Source: Oncotarget. 2017 Apr 21;8(30):49201–16. doi: 10.18632/oncotarget.17329 (PMC5564761; doi:10.18632/oncotarget.17329)
Supplement: Supplementary file 1 [file oncotarget-08-49201-s001.pdf]

Identification of aberrantly expressed long non-coding RNAs in stomach adenocarcinoma

Supplementary Materials

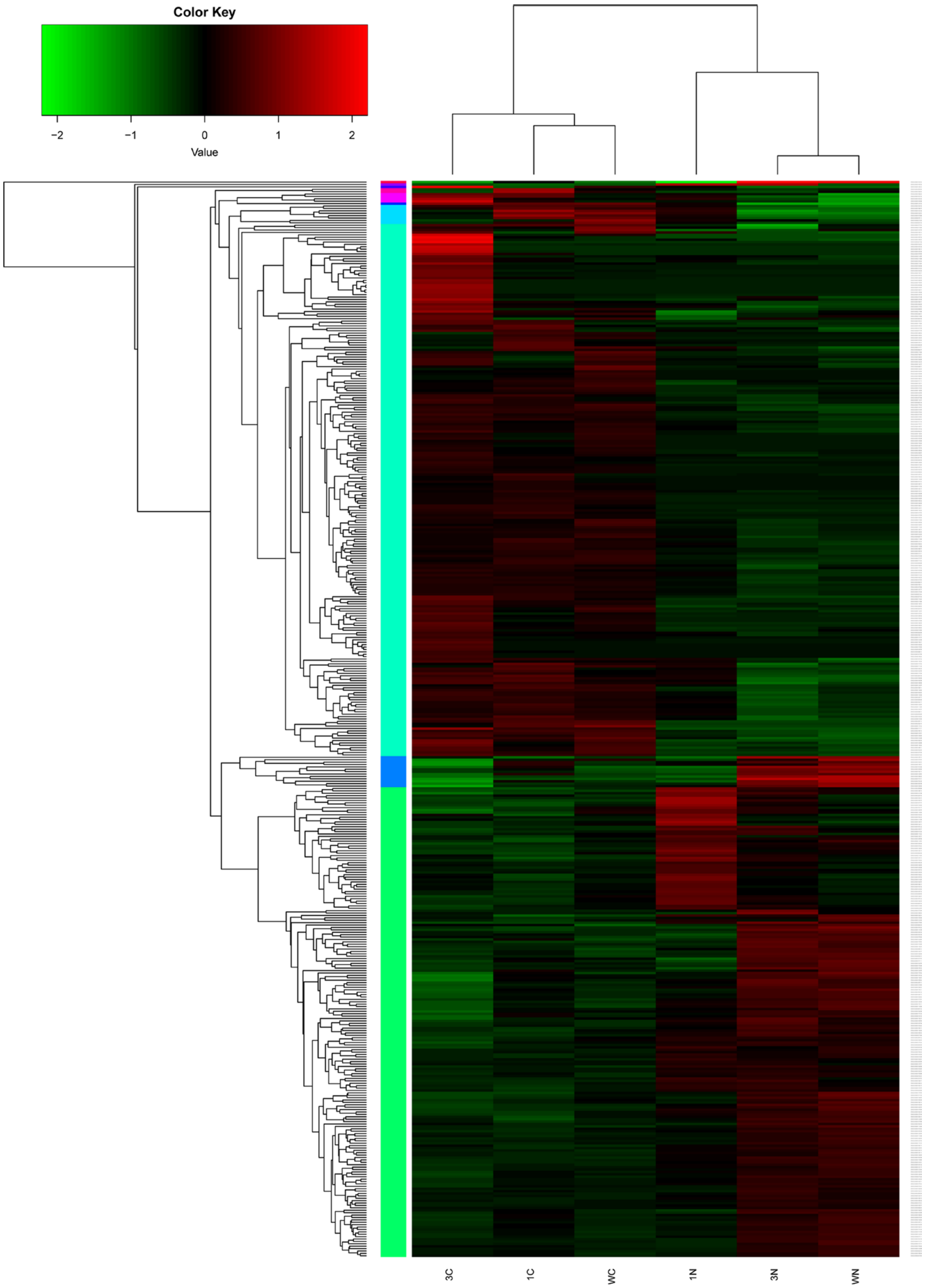

Supplementary Figure 1: Hierarchical clustering analysis of the expression levels of DEmRNAs in stomach adenocarcinoma and adjacent non-tumor tissues. Row and column represented DElncRNAs and tissue samples. The color scale indicated log_{10}FPKM of the expression level of DElncRNAs. Red and green indicated up- and down-regulated DElncRNAs. C represented stomach adenocarcinoma tissues and N represented adjacent non-tumor tissues.

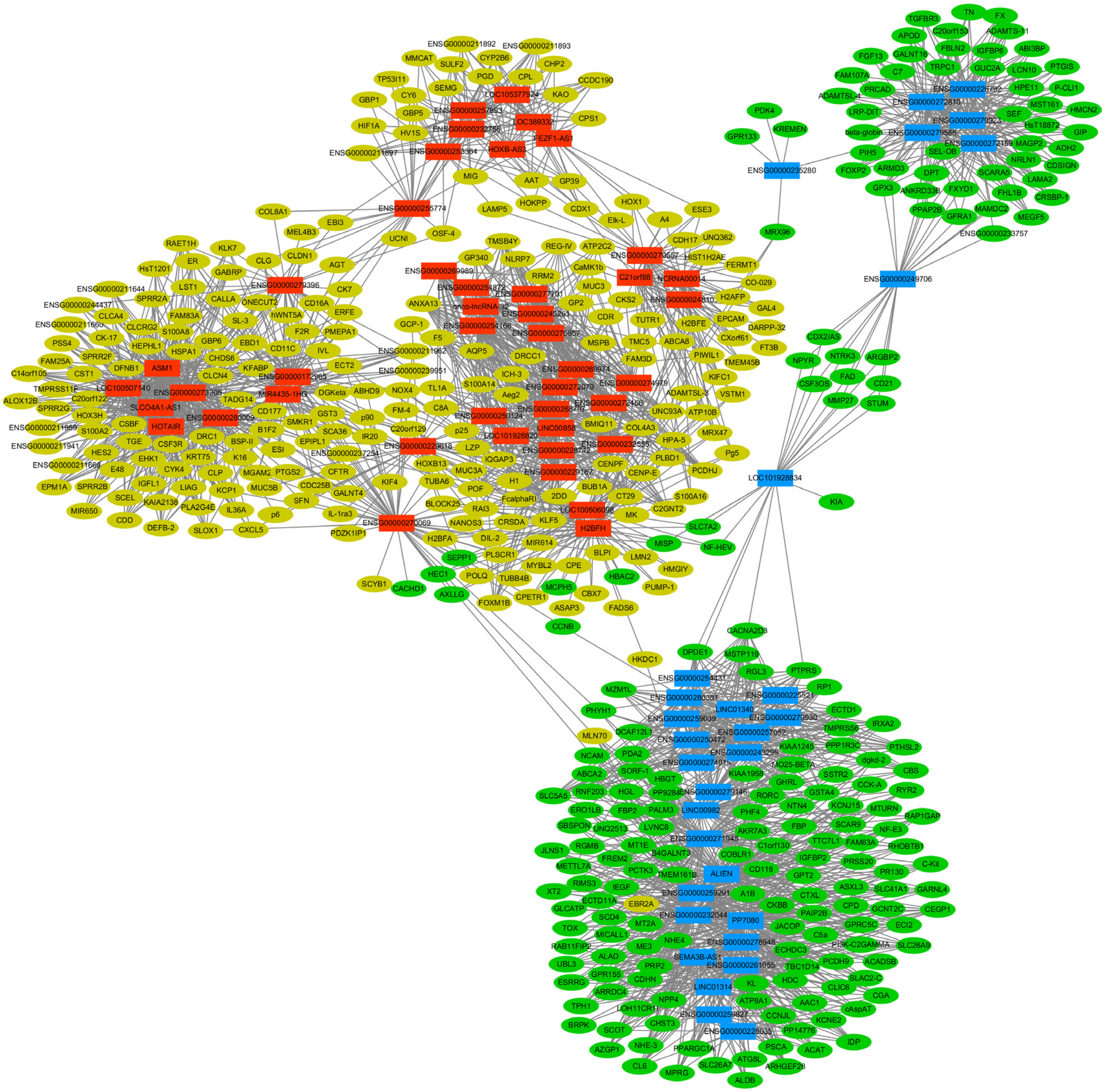

Supplementary Figure 2: Co-expression network of 445 DEmRNAs and 74 DElncRNAs. Circular node represented DEmRNA and rectangle node represented DElncRNA. Yellow was up-regulated DEmRNA and green was down-regulated DEmRNAs; Red indicated up-regulated DElncRNA and blue indicated down-regulated DElncRNA.

Supplementary Table 1: The basic information of patients with stomach adenocarcinoma

Number	Gender	Age	Lauren type	Histological type	TNM stage
1	Male	65	Diffuse	adenocarcinoma	T4N1M0
2	Male	58	Diffuse	adenocarcinoma	T4N1M0
3	Female	68	Diffuse	adenocarcinoma	T4N1M0

Supplementary Table 2: The raw sequence of high-throughput sequencing of samples

Samples	Clean reads	Base pairs	Error%	Q20%	Q30%	GC%
1C	104874284	12866223182	0.0278	97.05	93.57	45.48
1N	103724778	12638270038	0.0303	96.08	91.73	45.52
3C	94596168	11583136450	0.0282	96.87	93.21	45.1
3N	88640770	10875400402	0.028	96.97	93.38	48.08
WC	115223050	14107115777	0.0285	96.76	92.98	45.61
WN	98382204	12066513948	0.0283	96.87	93.18	47.64

C indicated stomach adenocarcinoma tissues; N indicated adjacent non-tumor tissue; Q20: the percentage of bases with quality value more than 20; Q30: the percentage of bases with quality value more than 30.

Supplementary Table 3: The mapped ratio of all samples

Samples	Total Reads	Mapped Reads	Mapped Ratio	Uniq Mapped Reads	Uniq Mapped Ratio
1C	104874284	73819118	70.39%	63598082	60.64%
1N	103724778	75167965	72.47%	61240981	59.04%
2C	94596168	73519289	77.72%	63086510	66.69%
2N	88640770	71531644	80.70%	50968434	57.50%
3C	115223050	94134136	81.70%	81886434	71.07%
3N	98382204	78094634	79.38%	52710475	53.58%

C indicated stomach adenocarcinoma samples; N indicated matched adjacent non-tumor tissues.

Supplementary Table 4: The full list of DElncRNAs in STAD. See Supplementary_Table_4

Supplementary Table 5: The full list of DEmRNAs in STAD. See Supplementary_Table_5

Supplementary Table 6: The primers of DElnRNAs and DEmRNAs for qRT-PCR verification

Gene	Primers
SNHG3	Forward- AGACAGATTCGCAGTGGTCG
	Reverse- GTCTCCATGGCCCACTTCTG
ENSG00000177133	Forward-TTTCTGGCACCAGAGATTGAG
	Reverse-CATCCAGGCAACAGCATTCG
FOXA2	Forward-TGCACTCGGCTTCCAGTATG
	Reverse-CATGTTGCTCACGGAGGAGT
LINC00261	Forward-CAGATTCAGTGACAC ATTC
	Reverse-TGCCTTTCTTTGCTCCAAT
β-actin	Forward-AGCGAGCATCCCCCAAAGTT
	Reverse-GGGCACGAAGGCTCATCATT

DElncRNAs: differentially expressed lncRNAs; DEmRNA: differentially expressed mRNAs.
