# Supplementary material for: Identification of aberrantly expressed long non-coding RNAs in stomach adenocarcinoma
Source: Oncotarget. 2017 Apr 21;8(30):49201–16. doi: 10.18632/oncotarget.17329 (PMC5564761; doi:10.18632/oncotarget.17329)
Supplement: Supplementary file 2 [file oncotarget-08-49201-s002.docx]

**Supplementary Table 4: The full list of DElncRNAs in STAD**

| **Ensembl Gene ID** | **Gene Symbol** | **FDR** | **log_2_FC** |
| --- | --- | --- | --- |
| **Up-regulation** | | | |
| ENSG00000282408 | LOC105377924 | 0.00238636 | 8.040191072 |
| ENSG00000229618 | AC011288.2 | 0.00238636 | 6.394160833 |
| ENSG00000254166 | CASC19 | 0.00238636 | 6.274222518 |
| ENSG00000229167 | RP11-73M7.1 | 0.00238636 | 6.002958997 |
| ENSG00000250124 | CTC-261N6.2 | 0.00238636 | 5.666884384 |
| ENSG00000269989 | RP11-635N19.3 | 0.00238636 | 5.654653212 |
| ENSG00000272468 | RP1-86C11.7 | 0.00238636 | 5.428778891 |
| ENSG00000248103 | CTC-338M12.9 | 0.00238636 | 5.305211776 |
| ENSG00000273165 | RP11-1057B6.1 | 0.00820313 | 5.254068658 |
| ENSG00000230316 | FEZF1-AS1 | 0.00238636 | 5.249581867 |
| ENSG00000268707 | RP11-247A12.7 | 0.004375 | 5.209936782 |
| ENSG00000232803 | SLCO4A1-AS1 | 0.00238636 | 5.176534214 |
| ENSG00000275857 | AC009133.21 | 0.00238636 | 5.173934925 |
| ENSG00000249550 | LINC01234 | 0.00238636 | 5.157593512 |
| ENSG00000269974 | RP11-932O9.10 | 0.00238636 | 5.095658636 |
| ENSG00000273305 | RP11-440D17.4 | 0.00238636 | 5.050858627 |
| ENSG00000228742 | [RP5-884M6.1](http://asia.ensembl.org/Homo_sapiens/Gene/Summary?g=ENSG00000228742&db=core) | 0.00238636 | 4.991435679 |
| ENSG00000232555 | [AC104088.1](http://asia.ensembl.org/Homo_sapiens/Gene/Summary?g=ENSG00000232555&db=core) | 0.00635081 | 4.948413958 |
| ENSG00000233834 | [AC005083.1](http://asia.ensembl.org/Homo_sapiens/Gene/Summary?g=ENSG00000233834&db=core) | 0.0238636 | 4.842466006 |
| ENSG00000255774 | [AP000439.3](http://asia.ensembl.org/Homo_sapiens/Gene/Summary?g=ENSG00000255774&db=core) | 0.00238636 | 4.768004308 |
| ENSG00000232756 | [RP5-1185I7.1](http://asia.ensembl.org/Homo_sapiens/Gene/Summary?g=ENSG00000232756&db=core) | 0.00238636 | 4.736723923 |
| ENSG00000130600 | [H19](http://asia.ensembl.org/Homo_sapiens/Gene/Summary?g=ENSG00000130600&db=core) | 0.00238636 | 4.554142313 |
| ENSG00000237166 | LINC01792 | 0.00238636 | 4.368530906 |
| ENSG00000234155 | RP11-30P6.6 | 0.00238636 | 4.355446245 |
| ENSG00000272079 | [LA16c-380H5.5](http://asia.ensembl.org/Homo_sapiens/Gene/Summary?g=ENSG00000272079&db=core) | 0.00238636 | 4.343287 |
| ENSG00000229404 | [LINC00858](http://asia.ensembl.org/Homo_sapiens/Gene/Summary?g=ENSG00000229404&db=core) | 0.00238636 | 4.330357613 |
| ENSG00000245261 | [RP3-330M21.5](http://asia.ensembl.org/Homo_sapiens/Gene/Summary?g=ENSG00000245261&db=core) | 0.00238636 | 4.264521418 |
| ENSG00000254872 | [RP13-870H17.3](http://asia.ensembl.org/Homo_sapiens/Gene/Summary?g=ENSG00000254872&db=core) | 0.00820312 | 4.225458333 |
| ENSG00000280009 | [CTD-2123J17.2](http://asia.ensembl.org/Homo_sapiens/Gene/Summary?g=ENSG00000280009&db=core) | 0.00238636 | 4.13325937 |
| ENSG00000228630 | HOTAIR | 0.00238636 | 4.132190208 |
| ENSG00000279396 | [AC130469.1](http://asia.ensembl.org/Homo_sapiens/Gene/Summary?g=ENSG00000279396&db=core) | 0.004375 | 4.105451801 |
| ENSG00000257893 | [RP11-587P21.2](http://asia.ensembl.org/Homo_sapiens/Gene/Summary?g=ENSG00000257893&db=core) | 0.00238636 | 3.987393636 |
| ENSG00000271824 | LOC389332 | 0.004375 | 3.903028626 |
| ENSG00000270557 | [RP11-546J1.1](http://asia.ensembl.org/Homo_sapiens/Gene/Summary?g=ENSG00000270557&db=core) | 0.004375 | 3.838195439 |
| ENSG00000233968 | [RP11-354E11.2](http://asia.ensembl.org/Homo_sapiens/Gene/Summary?g=ENSG00000233968&db=core) | 0.00238636 | 3.72799667 |
| ENSG00000184809 | B3GALT5-AS1 | 0.00238636 | 3.611113349 |
| ENSG00000233101 | HOXB-AS3 | 0.00238636 | 3.493317546 |
| ENSG00000277701 | [RP11-734K23.9](http://asia.ensembl.org/Homo_sapiens/Gene/Summary?g=ENSG00000277701&db=core) | 0.0461149 | 3.361121474 |
| ENSG00000253364 | [RP11-731F5.2](http://asia.ensembl.org/Homo_sapiens/Gene/Summary?g=ENSG00000253364&db=core) | 0.00635081 | 3.258530507 |
| ENSG00000172965 | [MIR4435-2HG](http://asia.ensembl.org/Homo_sapiens/Gene/Summary?g=ENSG00000172965&db=core) | 0.0121154 | 2.751083178 |
| ENSG00000274979 | [RP11-1143G9.5](http://asia.ensembl.org/Homo_sapiens/Gene/Summary?g=ENSG00000274979&db=core) | 0.0401042 | 1.912882572 |
| ENSG00000270069 | [MIR222HG](http://asia.ensembl.org/Homo_sapiens/Gene/Summary?g=ENSG00000270069&db=core) | 0.0289522 | 1.87417519 |
| ENSG00000242125 | SNHG3 | 0.0304348 | 1.730205788 |
| **Down-regulation** | | | |
| ENSG00000232352 | SEMA3B-AS1 | 0.00238636 | -6.99292701 |
| ENSG00000259417 | LINC01314 | 0.00238636 | -6.006864475 |
| ENSG00000272159 | RP11-350N15.6 | 0.00238636 | -5.912151637 |
| ENSG00000272810 | U91328.22 | 0.004375 | -5.801065509 |
| ENSG00000259827 | RP11-343H19.2 | 0.00238636 | -5.593494209 |
| ENSG00000225521 | AC005237.4 | 0.00238636 | -5.526766822 |
| ENSG00000274015 | CTD-2302E22.6 | 0.00238636 | -5.401633879 |
| ENSG00000259039 | RP11-409I10.2 | 0.00238636 | -5.179666299 |
| ENSG00000250331 | LINC01340 | 0.00238636 | -5.097315823 |
| ENSG00000250472 | TRIM36-IT1 | 0.00238636 | -5.061832349 |
| ENSG00000264754 | CTD-2653B5.1 | 0.00238636 | -5.041799308 |
| ENSG00000232044 | LINC01105 | 0.0461149 | -5.001686931 |
| ENSG00000177133 | LINC00982 | 0.00238636 | -4.981227991 |
| ENSG00000257052 | RP11-881M11.2 | 0.00238636 | -4.971847005 |
| ENSG00000254431 | RP11-550A5.2 | 0.00238636 | -4.806200304 |
| ENSG00000243295 | [RP11-550A5.2](http://asia.ensembl.org/Homo_sapiens/Gene/Summary?g=ENSG00000254431&db=core) | 0.00238636 | -4.626234692 |
| ENSG00000235280 | [MCF2L-AS1](http://asia.ensembl.org/Homo_sapiens/Gene/Summary?g=ENSG00000235280&db=core) | 0.00238636 | -4.620469113 |
| ENSG00000279586 | [AP000711.1](http://asia.ensembl.org/Homo_sapiens/Gene/Summary?g=ENSG00000279586&db=core) | 0.00238636 | -4.610292564 |
| ENSG00000226792 | [LINC00371](http://asia.ensembl.org/Homo_sapiens/Gene/Summary?g=ENSG00000226792&db=core) | 0.00238636 | -4.368216584 |
| ENSG00000271945 | [RP11-354K4.2](http://asia.ensembl.org/Homo_sapiens/Gene/Summary?g=ENSG00000271945&db=core) | 0.00238636 | -4.286555913 |
| ENSG00000249706 | [RP11-89B16.1](http://asia.ensembl.org/Homo_sapiens/Gene/Summary?g=ENSG00000249706&db=core) | 0.00238636 | -4.260281644 |
| ENSG00000279146 | [RP11-117L5.3](http://asia.ensembl.org/Homo_sapiens/Gene/Summary?g=ENSG00000279146&db=core) | 0.00238636 | -4.003386356 |
| ENSG00000279923 | [CTD-2008E3.1](http://asia.ensembl.org/Homo_sapiens/Gene/Summary?g=ENSG00000279923&db=core) | 0.00238636 | -3.944286875 |
| ENSG00000279930 | [LA16c-312E8.4](http://asia.ensembl.org/Homo_sapiens/Gene/Summary?g=ENSG00000279930&db=core) | 0.00238636 | -3.861042228 |
| ENSG00000228035 | [RP4-663N10.1](http://asia.ensembl.org/Homo_sapiens/Gene/Summary?g=ENSG00000228035&db=core) | 0.00238636 | -3.716003161 |
| ENSG00000261055 | [RP11-195M16.3](http://asia.ensembl.org/Homo_sapiens/Gene/Summary?g=ENSG00000261055&db=core) | 0.00238636 | -3.663663412 |
| ENSG00000259291 | [RP11-617F23.1](http://asia.ensembl.org/Homo_sapiens/Gene/Summary?g=ENSG00000259291&db=core) | 0.00238636 | -2.886146329 |
| ENSG00000278948 | [RP5-1039K5.12](http://asia.ensembl.org/Homo_sapiens/Gene/Summary?g=ENSG00000278948&db=core) | 0.00238636 | -2.704312409 |
| ENSG00000259974 | LINC00261 | 0.0332746 | -2.676333929 |
| ENSG00000280381 | [RP11-197N18.8](http://asia.ensembl.org/Homo_sapiens/Gene/Summary?g=ENSG00000280381&db=core) | 0.031875 | -2.066660802 |
| ENSG00000188242 | PP7080 | 0.0289522 | -1.728634992 |

DElncRNAs: differentially expressed long non-coding RNA; STAD: stomach adenocarcinoma; FDR: false discovery rate; FC: fold change.
