# Supplementary material for: Identification of aberrantly expressed long non-coding RNAs in stomach adenocarcinoma
Source: Oncotarget. 2017 Apr 21;8(30):49201–16. doi: 10.18632/oncotarget.17329 (PMC5564761; doi:10.18632/oncotarget.17329)
Supplement: Supplementary file 3 [file oncotarget-08-49201-s003.docx]

**Supplementary Table 5: The full list of DEmRNAs in STAD**

| **Ensembl Gene ID** | **Gene Symbol** | **FDR** | **log_2_FC** | |
| --- | --- | --- | --- | --- |
| **Up-regulation** | | | | |
| ENSG00000196754 | S100A2 | 0.00337364 | | 7.183158953 |
| ENSG00000186832 | KRT16 | 0.00337364 | | 6.789708057 |
| ENSG00000128422 | KRT17 | 0.0262905 | | 6.716559208 |
| ENSG00000241794 | SPRR2A | 0.00337364 | | 6.546179879 |
| ENSG00000170373 | CST1 | 0.00337364 | | 6.349039672 |
| ENSG00000143546 | S100A8 | 0.00337364 | | 5.623496687 |
| ENSG00000119547 | ONECUT2 | 0.00337364 | | 5.255570479 |
| ENSG00000188100 | FAM25A | 0.00337364 | | 5.221544589 |
| ENSG00000159516 | SPRR2G | 0.00337364 | | 4.984393393 |
| ENSG00000171711 | DEFB4A | 0.00337364 | | 4.874560923 |
| ENSG00000143536 | CRNN | 0.00337364 | | 4.823932742 |
| ENSG00000125780 | TGM3 | 0.00830821 | | 4.667539055 |
| ENSG00000149968 | MMP3 | 0.00337364 | | 4.616511381 |
| ENSG00000163347 | CLDN1 | 0.0198214 | | 4.494385576 |
| ENSG00000165474 | GJB2 | 0.00337364 | | 4.389336874 |
| ENSG00000124102 | PI3 | 0.0161348 | | 4.388491092 |
| ENSG00000169347 | GP2 | 0.00337364 | | 4.321979183 |
| ENSG00000156510 | HKDC1 | 0.0262905 | | 4.145071874 |
| ENSG00000099953 | MMP11 | 0.00830821 | | 4.132132617 |
| ENSG00000163207 | IVL | 0.00337364 | | 4.130584977 |
| ENSG00000167656 | LY6D | 0.00337364 | | 4.106591205 |
| ENSG00000211660 | IGLV2-23 | 0.0363824 | | 4.066036638 |
| ENSG00000257743 | MGAM2 | 0.00337364 | | 4.04319901 |
| ENSG00000113722 | CDX1 | 0.00337364 | | 4.027941373 |
| ENSG00000187908 | DMBT1 | 0.00337364 | | 3.984090814 |
| ENSG00000137673 | MMP7 | 0.0280832 | | 3.969376161 |
| ENSG00000169035 | KLK7 | 0.00337364 | | 3.908784092 |
| ENSG00000173391 | OLR1 | 0.00337364 | | 3.891529787 |
| ENSG00000263639 | MSMB | 0.00337364 | | 3.696697453 |
| ENSG00000133048 | CHI3L1 | 0.00337364 | | 3.692095703 |
| ENSG00000211941 | IGHV3-11 | 0.0418141 | | 3.652374424 |
| ENSG00000256713 | PGA5 | 0.00337364 | | 3.623515741 |
| ENSG00000163220 | S100A9 | 0.00337364 | | 3.585296381 |
| ENSG00000001626 | CFTR | 0.00337364 | | 3.58082884 |
| ENSG00000079112 | CDH17 | 0.00598548 | | 3.548181716 |
| ENSG00000135480 | KRT7 | 0.00337364 | | 3.51829881 |
| ENSG00000134193 | REG4 | 0.00337364 | | 3.499492166 |
| ENSG00000102837 | OLFM4 | 0.00337364 | | 3.434533016 |
| ENSG00000273703 | HIST1H2BM | 0.0338218 | | 3.395981051 |
| ENSG00000204936 | CD177 | 0.043051 | | 3.343012356 |
| ENSG00000163735 | CXCL5 | 0.00337364 | | 3.318731519 |
| ENSG00000106031 | HOXA13 | 0.00337364 | | 3.283524754 |
| ENSG00000154620 | TMSB4Y | 0.00337364 | | 3.281573631 |
| ENSG00000138755 | CXCL9 | 0.00337364 | | 3.278246588 |
| ENSG00000196611 | MMP1 | 0.00337364 | | 3.254510612 |
| ENSG00000167768 | KRT1 | 0.00337364 | | 3.23968979 |
| ENSG00000138315 | OIT3 | 0.00337364 | | 3.213813846 |
| ENSG00000204019 | CT83 | 0.00337364 | | 3.21251798 |
| ENSG00000137975 | CLCA2 | 0.00337364 | | 3.209575015 |
| ENSG00000244094 | SPRR2F | 0.00337364 | | 3.190080664 |
| ENSG00000183347 | GBP6 | 0.0407413 | | 3.173707346 |
| ENSG00000172782 | FADS6 | 0.00830821 | | 3.161649094 |
| ENSG00000167755 | KLK6 | 0.00337364 | | 3.156673285 |
| ENSG00000157131 | C8A | 0.00337364 | | 3.144997517 |
| ENSG00000116833 | NR5A2 | 0.0178306 | | 3.092500255 |
| ENSG00000198610 | AKR1C4 | 0.00598548 | | 3.079956609 |
| ENSG00000121552 | CSTA | 0.00337364 | | 3.058872682 |
| ENSG00000002726 | AOC1 | 0.00337364 | | 3.032140096 |
| ENSG00000211892 | IGHG4 | 0.00337364 | | 3.031505626 |
| ENSG00000196549 | MME | 0.00337364 | | 3.018449829 |
| ENSG00000136694 | IL36A | 0.00337364 | | 3.004042893 |
| ENSG00000117983 | MUC5B | 0.00337364 | | 2.979443357 |
| ENSG00000136155 | SCEL | 0.00337364 | | 2.97004191 |
| ENSG00000188373 | C10orf99 | 0.00337364 | | 2.942966713 |
| ENSG00000189143 | CLDN4 | 0.00830821 | | 2.91266468 |
| ENSG00000203747 | FCGR3A | 0.00337364 | | 2.826395346 |
| ENSG00000169429 | CXCL8 | 0.00337364 | | 2.801539172 |
| ENSG00000196805 | SPRR2B | 0.00337364 | | 2.735448524 |
| ENSG00000021826 | CPS1 | 0.0262905 | | 2.735327244 |
| ENSG00000104537 | ANXA13 | 0.00337364 | | 2.696628192 |
| ENSG00000278196 | IGLV2-8 | 0.00598548 | | 2.69406894 |
| ENSG00000148346 | LCN2 | 0.00337364 | | 2.68485325 |
| ENSG00000013588 | GPRC5A | 0.00598548 | | 2.67118376 |
| ENSG00000170454 | KRT75 | 0.00337364 | | 2.616098873 |
| ENSG00000164694 | FNDC1 | 0.00337364 | | 2.608132624 |
| ENSG00000114251 | WNT5A | 0.00337364 | | 2.602097685 |
| ENSG00000164611 | PTTG1 | 0.0338218 | | 2.587631427 |
| ENSG00000181634 | TNFSF15 | 0.00337364 | | 2.583785055 |
| ENSG00000101057 | MYBL2 | 0.00337364 | | 2.566193058 |
| ENSG00000110492 | MDK | 0.00337364 | | 2.558726176 |
| ENSG00000268104 | SLC6A14 | 0.00830821 | | 2.5467862 |
| ENSG00000140297 | GCNT3 | 0.00337364 | | 2.514574881 |
| ENSG00000089472 | HEPH | 0.00337364 | | 2.459475562 |
| ENSG00000169894 | MUC3A | 0.00598548 | | 2.450689723 |
| ENSG00000163507 | KIAA1524 | 0.0246715 | | 2.42880045 |
| ENSG00000163221 | S100A12 | 0.00337364 | | 2.399135539 |
| ENSG00000080986 | NDC80 | 0.0214509 | | 2.389526651 |
| ENSG00000211893 | IGHG2 | 0.00830821 | | 2.382879129 |
| ENSG00000129455 | KLK8 | 0.00337364 | | 2.362688696 |
| ENSG00000244437 | IGKV3-15 | 0.00337364 | | 2.356239274 |
| ENSG00000051341 | POLQ | 0.00337364 | | 2.352251293 |
| ENSG00000161798 | AQP5 | 0.0246715 | | 2.33664141 |
| ENSG00000123975 | CKS2 | 0.0363824 | | 2.336279241 |
| ENSG00000204482 | LST1 | 0.0349921 | | 2.324752499 |
| ENSG00000144810 | COL8A1 | 0.0178306 | | 2.322300252 |
| ENSG00000101447 | FAM83D | 0.0122881 | | 2.313542955 |
| ENSG00000170477 | KRT4 | 0.00337364 | | 2.283263967 |
| ENSG00000088325 | TPX2 | 0.00337364 | | 2.280707122 |
| ENSG00000124107 | SLPI | 0.00337364 | | 2.277204891 |
| ENSG00000154451 | GBP5 | 0.00337364 | | 2.27071866 |
| ENSG00000175538 | KCNE3 | 0.0104168 | | 2.268010534 |
| ENSG00000073464 | CLCN4 | 0.0326031 | | 2.265698474 |
| ENSG00000169876 | MUC17 | 0.0122881 | | 2.26160723 |
| ENSG00000197410 | DCHS2 | 0.0214509 | | 2.235161801 |
| ENSG00000073756 | PTGS2 | 0.0178306 | | 2.226332673 |
| ENSG00000211962 | IGHV1-46 | 0.00337364 | | 2.206418176 |
| ENSG00000084207 | GSTP1 | 0.00337364 | | 2.203073648 |
| ENSG00000090104 | RGS1 | 0.00337364 | | 2.192494546 |
| ENSG00000114270 | COL7A1 | 0.00337364 | | 2.186919948 |
| ENSG00000094755 | GABRP | 0.00598548 | | 2.166333571 |
| ENSG00000131771 | PPP1R1B | 0.0338218 | | 2.163415852 |
| ENSG00000136689 | IL1RN | 0.00337364 | | 2.156973558 |
| ENSG00000189334 | S100A14 | 0.00337364 | | 2.156934705 |
| ENSG00000148773 | MKI67 | 0.00337364 | | 2.153677861 |
| ENSG00000164687 | FABP5 | 0.00337364 | | 2.147301416 |
| ENSG00000168703 | WFDC12 | 0.00337364 | | 2.133688457 |
| ENSG00000114346 | ECT2 | 0.00337364 | | 2.122939727 |
| ENSG00000134057 | CCNB1 | 0.0455441 | | 2.107420906 |
| ENSG00000188293 | IGFL1 | 0.00337364 | | 2.100412566 |
| ENSG00000173702 | MUC13 | 0.00337364 | | 2.100214286 |
| ENSG00000211959 | IGHV4-39 | 0.0455441 | | 2.098422036 |
| ENSG00000171747 | LGALS4 | 0.00337364 | | 2.085774595 |
| ENSG00000118322 | ATP10B | 0.0142731 | | 2.07800392 |
| ENSG00000066279 | ASPM | 0.00337364 | | 2.071530344 |
| ENSG00000273802 | HIST1H2BG | 0.00830821 | | 2.05382062 |
| ENSG00000111206 | FOXM1 | 0.0104168 | | 2.010662141 |
| ENSG00000100557 | C14orf105 | 0.00337364 | | 2.010310797 |
| ENSG00000090889 | KIF4A | 0.0198214 | | 2.002407308 |
| ENSG00000171848 | RRM2 | 0.0104168 | | 1.986741831 |
| ENSG00000138778 | CENPE | 0.00337364 | | 1.982985312 |
| ENSG00000274290 | HIST1H2BE | 0.0233263 | | 1.968890861 |
| ENSG00000175793 | SFN | 0.0104168 | | 1.955540325 |
| ENSG00000196562 | SULF2 | 0.0338218 | | 1.953145367 |
| ENSG00000211644 | IGLV1-51 | 0.0122881 | | 1.932854207 |
| ENSG00000211666 | IGLV2-14 | 0.0246715 | | 1.931322128 |
| ENSG00000197249 | SERPINA1 | 0.00337364 | | 1.929994944 |
| ENSG00000064270 | ATP2C2 | 0.0122881 | | 1.917564261 |
| ENSG00000183856 | IQGAP3 | 0.00337364 | | 1.915064459 |
| ENSG00000121316 | PLBD1 | 0.0483681 | | 1.906321815 |
| ENSG00000146039 | SLC17A4 | 0.00337364 | | 1.880669854 |
| ENSG00000127324 | TSPAN8 | 0.00337364 | | 1.879665524 |
| ENSG00000198734 | F5 | 0.039251 | | 1.876830028 |
| ENSG00000112378 | PERP | 0.00337364 | | 1.874271647 |
| ENSG00000277075 | HIST1H2AE | 0.00337364 | | 1.863362156 |
| ENSG00000163739 | CXCL1 | 0.0246715 | | 1.855783072 |
| ENSG00000135744 | AGT | 0.0262905 | | 1.832041221 |
| ENSG00000171124 | FUT3 | 0.0338218 | | 1.830418681 |
| ENSG00000184357 | HIST1H1B | 0.00337364 | | 1.798875476 |
| ENSG00000237254 | TRBV30 | 0.00337364 | | 1.796460704 |
| ENSG00000211897 | IGHG3 | 0.00830821 | | 1.794463907 |
| ENSG00000124225 | PMEPA1 | 0.00337364 | | 1.782371821 |
| ENSG00000164171 | ITGA2 | 0.0122881 | | 1.77499597 |
| ENSG00000196787 | HIST1H2AG | 0.0443614 | | 1.772535795 |
| ENSG00000101311 | FERMT1 | 0.00598548 | | 1.750905166 |
| ENSG00000119535 | CSF3R | 0.0363824 | | 1.719613342 |
| ENSG00000151715 | TMEM45B | 0.0104168 | | 1.699294937 |
| ENSG00000181333 | HEPHL1 | 0.00337364 | | 1.691793201 |
| ENSG00000102780 | DGKH | 0.0214509 | | 1.678929935 |
| ENSG00000117724 | CENPF | 0.00337364 | | 1.673271807 |
| ENSG00000160213 | CSTB | 0.0349921 | | 1.662946614 |
| ENSG00000090776 | EFNB1 | 0.00337364 | | 1.660317816 |
| ENSG00000237649 | KIFC1 | 0.0142731 | | 1.648769821 |
| ENSG00000099812 | MISP | 0.039251 | | 1.646620567 |
| ENSG00000257594 | GALNT4 | 0.043051 | | 1.644133273 |
| ENSG00000142657 | PGD | 0.0122881 | | 1.639963591 |
| ENSG00000103534 | TMC5 | 0.00337364 | | 1.635781334 |
| ENSG00000135373 | EHF | 0.0298472 | | 1.6053686 |
| ENSG00000162366 | PDZK1IP1 | 0.00598548 | | 1.597122621 |
| ENSG00000140678 | ITGAX | 0.039251 | | 1.590338968 |
| ENSG00000169679 | BUB1 | 0.0363824 | | 1.586424775 |
| ENSG00000058085 | LAMC2 | 0.031333 | | 1.585610877 |
| ENSG00000110400 | NECTIN1 | 0.0122881 | | 1.584695943 |
| ENSG00000113368 | LMNB1 | 0.0379226 | | 1.571963403 |
| ENSG00000175274 | TP53I11 | 0.0326031 | | 1.570486227 |
| ENSG00000124429 | POF1B | 0.0214509 | | 1.543922989 |
| ENSG00000139618 | BRCA2 | 0.039251 | | 1.542596807 |
| ENSG00000137812 | KNL1 | 0.0161348 | | 1.536426376 |
| ENSG00000137309 | HMGA1 | 0.00598548 | | 1.52577313 |
| ENSG00000167553 | TUBA1C | 0.0122881 | | 1.519693177 |
| ENSG00000198643 | FAM3D | 0.0349921 | | 1.511963026 |
| ENSG00000101224 | CDC25B | 0.0326031 | | 1.506562806 |
| ENSG00000140937 | CDH11 | 0.031333 | | 1.506027402 |
| ENSG00000188643 | S100A16 | 0.0104168 | | 1.499987846 |
| ENSG00000197632 | SERPINB2 | 0.0443614 | | 1.499006489 |
| ENSG00000102007 | PLP2 | 0.0142731 | | 1.489773026 |
| ENSG00000124233 | SEMG1 | 0.00337364 | | 1.489558349 |
| ENSG00000261150 | EPPK1 | 0.0161348 | | 1.48261073 |
| ENSG00000239951 | IGKV3-20 | 0.0214509 | | 1.471690813 |
| ENSG00000117228 | GBP1 | 0.00337364 | | 1.471263017 |
| ENSG00000188229 | TUBB4B | 0.00830821 | | 1.462069206 |
| ENSG00000143891 | GALM | 0.0280832 | | 1.45707021 |
| ENSG00000096006 | CRISP3 | 0.00337364 | | 1.456874472 |
| ENSG00000158825 | CDA | 0.00337364 | | 1.455365363 |
| ENSG00000160932 | LY6E | 0.031333 | | 1.452827382 |
| ENSG00000101361 | NOP56 | 0.00337364 | | 1.447302448 |
| ENSG00000188089 | PLA2G4E | 0.00337364 | | 1.44330429 |
| ENSG00000188313 | PLSCR1 | 0.00830821 | | 1.436044814 |
| ENSG00000181104 | F2R | 0.0349921 | | 1.401531486 |
| ENSG00000137757 | CASP5 | 0.00337364 | | 1.363490706 |
| ENSG00000125207 | PIWIL1 | 0.00337364 | | 1.349314318 |
| ENSG00000119888 | EPCAM | 0.0214509 | | 1.348649605 |
| ENSG00000186431 | FCAR | 0.00337364 | | 1.347824372 |
| ENSG00000204389 | HSPA1A | 0.0483681 | | 1.333857017 |
| ENSG00000102554 | KLF5 | 0.0233263 | | 1.332774432 |
| ENSG00000100644 | HIF1A | 0.0495902 | | 1.273534898 |
| ENSG00000163191 | S100A11 | 0.0495902 | | 1.155504152 |
| ENSG00000147689 | FAM83A | 0.00337364 | | 1.126920009 |
| ENSG00000132911 | NMUR2 | 0.00337364 | | 1.100735497 |
| ENSG00000179477 | ALOX12B | 0.00337364 | | 0.968031752 |
| ENSG00000108342 | CSF3 | 0.00337364 | | 0.806258057 |
| ENSG00000165556 | CDX2 | 0.00337364 | | 0.763802473 |
| ENSG00000123388 | HOXC11 | 0.00337364 | | 0.76034019 |
| ENSG00000145040 | UCN2 | 0.00337364 | | 0.753707216 |
| ENSG00000178363 | CALML3 | 0.00337364 | | 0.641703237 |
| ENSG00000167634 | NLRP7 | 0.0178306 | | 0.63840753 |
| ENSG00000086991 | NOX4 | 0.00337364 | | 0.62181511 |
| ENSG00000105246 | EBI3 | 0.00337364 | | 0.606991722 |
| ENSG00000189068 | VSTM1 | 0.00337364 | | 0.587413 |
| ENSG00000029559 | IBSP | 0.00337364 | | 0.558110864 |
| ENSG00000187556 | NANOS3 | 0.00337364 | | 0.54842676 |
| ENSG00000130822 | PNCK | 0.00337364 | | 0.486765851 |
| ENSG00000178752 | ERFE | 0.00337364 | | 0.458791445 |
| ENSG00000104808 | DHDH | 0.00598548 | | 0.414168014 |
| ENSG00000131015 | ULBP2 | 0.00337364 | | 0.404652481 |
| ENSG00000198092 | TMPRSS11F | 0.00337364 | | 0.38084025 |
| ENSG00000140873 | ADAMTS18 | 0.00337364 | | 0.334110568 |
| ENSG00000179934 | CCR8 | 0.00337364 | | 0.255331528 |
| ENSG00000125869 | LAMP5 | 0.00337364 | | 0.241449719 |
| ENSG00000124882 | EREG | 0.00337364 | | 0.218458811 |
| ENSG00000159184 | HOXB13 | 0.00337364 | | 0.215206421 |
| ENSG00000105131 | EPHX3 | 0.00337364 | | 0.189704395 |
| ENSG00000185860 | CCDC190 | 0.0142731 | | 0.169347808 |
| ENSG00000240204 | SMKR1 | 0.00337364 | | 0.134772936 |
| ENSG00000197408 | CYP2B6 | 0.00337364 | | 0.107848532 |
| ENSG00000016602 | CLCA4 | 0.00337364 | | 0.083615054 |
| ENSG00000166869 | CHP2 | 0.00337364 | | 0.068258062 |
| ENSG00000069812 | HES2 | 0.00337364 | | 0.029474089 |
| ENSG00000112494 | UNC93A | 0.0418141 | | 0.006951443 |
| **Down-regulation** | | | | |
| ENSG00000140538 | NTRK3 | 0.00337364 | -8.491899371 | |
| ENSG00000182333 | LIPF | 0.00337364 | -7.912516783 | |
| ENSG00000157017 | GHRL | 0.0104168 | -7.416490252 | |
| ENSG00000196482 | ESRRG | 0.00337364 | -6.641388726 | |
| ENSG00000164128 | NPY1R | 0.0455441 | -6.186723597 | |
| ENSG00000168079 | SCARA5 | 0.0470172 | -5.562468974 | |
| ENSG00000185615 | PDIA2 | 0.00337364 | -5.084124997 | |
| ENSG00000157445 | CACNA2D3 | 0.00337364 | -4.954236871 | |
| ENSG00000187045 | TMPRSS6 | 0.00337364 | -4.933712023 | |
| ENSG00000167779 | IGFBP6 | 0.00337364 | -4.816126842 | |
| ENSG00000166165 | CKB | 0.00337364 | -4.699316081 | |
| ENSG00000196616 | ADH1B | 0.00337364 | -4.664705946 | |
| ENSG00000159197 | KCNE2 | 0.00337364 | -4.534447847 | |
| ENSG00000159212 | CLIC6 | 0.00337364 | -4.51924504 | |
| ENSG00000100604 | CHGA | 0.00337364 | -4.462706299 | |
| ENSG00000147606 | SLC26A7 | 0.0104168 | -4.432064082 | |
| ENSG00000163394 | CCKAR | 0.00337364 | -4.371327677 | |
| ENSG00000110195 | FOLR1 | 0.00337364 | -4.360160741 | |
| ENSG00000133800 | LYVE1 | 0.00337364 | -4.357519964 | |
| ENSG00000168702 | LRP1B | 0.00337364 | -4.169334056 | |
| ENSG00000163815 | CLEC3B | 0.00337364 | -4.103810396 | |
| ENSG00000170561 | IRX2 | 0.0104168 | -4.087481182 | |
| ENSG00000164764 | SBSPON | 0.0379226 | -4.082993084 | |
| ENSG00000266964 | FXYD1 | 0.00830821 | -4.069264072 | |
| ENSG00000126218 | F10 | 0.00337364 | -4.061389139 | |
| ENSG00000187922 | LCN10 | 0.0483681 | -4.028524475 | |
| ENSG00000143196 | DPT | 0.00337364 | -3.929837431 | |
| ENSG00000163328 | GPR155 | 0.00337364 | -3.870402708 | |
| ENSG00000138615 | CILP | 0.00337364 | -3.854425532 | |
| ENSG00000109956 | B3GAT1 | 0.0418141 | -3.706398058 | |
| ENSG00000160862 | AZGP1 | 0.00337364 | -3.706380174 | |
| ENSG00000144331 | ZNF385B | 0.0214509 | -3.705899792 | |
| ENSG00000165072 | MAMDC2 | 0.00337364 | -3.698544268 | |
| ENSG00000186197 | EDARADD | 0.0122881 | -3.599659085 | |
| ENSG00000077264 | PAK3 | 0.00598548 | -3.567962206 | |
| ENSG00000197614 | MFAP5 | 0.00337364 | -3.48504661 | |
| ENSG00000104237 | RP1 | 0.0349921 | -3.463208872 | |
| ENSG00000105650 | PDE4C | 0.00337364 | -3.457091384 | |
| ENSG00000110436 | SLC1A2 | 0.00337364 | -3.450528767 | |
| ENSG00000135083 | CCNJL | 0.00830821 | -3.41651804 | |
| ENSG00000131386 | GALNT15 | 0.00598548 | -3.363230763 | |
| ENSG00000175356 | SCUBE2 | 0.00337364 | -3.352883805 | |
| ENSG00000170558 | CDH2 | 0.0407413 | -3.343832394 | |
| ENSG00000140287 | HDC | 0.00337364 | -3.281107688 | |
| ENSG00000129682 | FGF13 | 0.0122881 | -3.254512404 | |
| ENSG00000174502 | SLC26A9 | 0.00337364 | -3.247063516 | |
| ENSG00000187867 | PALM3 | 0.00598548 | -3.19659192 | |
| ENSG00000113594 | LIFR | 0.00337364 | -3.191078068 | |
| ENSG00000056487 | PHF21B | 0.00337364 | -3.177236935 | |
| ENSG00000144730 | IL17RD | 0.0142731 | -3.17455441 | |
| ENSG00000148288 | GBGT1 | 0.00598548 | -3.169390219 | |
| ENSG00000205517 | RGL3 | 0.00598548 | -3.156195233 | |
| ENSG00000141431 | ASXL3 | 0.00337364 | -3.13390586 | |
| ENSG00000137675 | MMP27 | 0.00337364 | -3.123616539 | |
| ENSG00000153823 | PID1 | 0.00598548 | -3.113442127 | |
| ENSG00000184226 | PCDH9 | 0.00337364 | -3.089507491 | |
| ENSG00000158813 | EDA | 0.00830821 | -3.086311374 | |
| ENSG00000128849 | CGNL1 | 0.00337364 | -3.078630189 | |
| ENSG00000124212 | PTGIS | 0.0246715 | -3.055035621 | |
| ENSG00000170011 | MYRIP | 0.00337364 | -3.038592306 | |
| ENSG00000124374 | PAIP2B | 0.00337364 | -3.027424279 | |
| ENSG00000151729 | SLC25A4 | 0.00337364 | -3.021595856 | |
| ENSG00000086619 | ERO1B | 0.0495902 | -3.009360777 | |
| ENSG00000053918 | KCNQ1 | 0.00337364 | -2.99211998 | |
| ENSG00000064309 | CDON | 0.00337364 | -2.990776762 | |
| ENSG00000134463 | ECHDC3 | 0.00337364 | -2.963328208 | |
| ENSG00000102003 | SYP | 0.0104168 | -2.957551977 | |
| ENSG00000167653 | PSCA | 0.0122881 | -2.937640481 | |
| ENSG00000244734 | HBB | 0.00337364 | -2.92701545 | |
| ENSG00000186687 | LYRM7 | 0.0122881 | -2.911107103 | |
| ENSG00000015532 | XYLT2 | 0.00337364 | -2.878672679 | |
| ENSG00000101938 | CHRDL1 | 0.0122881 | -2.856263588 | |
| ENSG00000117016 | RIMS3 | 0.0198214 | -2.854648853 | |
| ENSG00000180616 | SSTR2 | 0.0104168 | -2.807090154 | |
| ENSG00000133116 | KL | 0.0161348 | -2.788212061 | |
| ENSG00000111452 | ADGRD1 | 0.00337364 | -2.754699454 | |
| ENSG00000148600 | CDHR1 | 0.0455441 | -2.747640113 | |
| ENSG00000144935 | TRPC1 | 0.039251 | -2.737627372 | |
| ENSG00000105641 | SLC5A5 | 0.00337364 | -2.732055343 | |
| ENSG00000154175 | ABI3BP | 0.0161348 | -2.725736992 | |
| ENSG00000168309 | FAM107A | 0.00337364 | -2.662287075 | |
| ENSG00000122863 | CHST3 | 0.00337364 | -2.657471054 | |
| ENSG00000141338 | ABCA8 | 0.00337364 | -2.640381457 | |
| ENSG00000170412 | GPRC5C | 0.00337364 | -2.636270927 | |
| ENSG00000203685 | STUM | 0.0326031 | -2.594421025 | |
| ENSG00000157111 | TMEM171 | 0.0418141 | -2.589152044 | |
| ENSG00000170899 | GSTA4 | 0.00337364 | -2.577965655 | |
| ENSG00000185432 | METTL7A | 0.0122881 | -2.573237379 | |
| ENSG00000111846 | GCNT2 | 0.00337364 | -2.572937104 | |
| ENSG00000160200 | CBS | 0.0349921 | -2.567967676 | |
| ENSG00000100626 | GALNT16 | 0.0455441 | -2.557794165 | |
| ENSG00000165124 | SVEP1 | 0.00337364 | -2.54918648 | |
| ENSG00000129167 | TPH1 | 0.031333 | -2.541231428 | |
| ENSG00000130957 | FBP2 | 0.0349921 | -2.53376034 | |
| ENSG00000109794 | FAM149A | 0.00598548 | -2.521838538 | |
| ENSG00000179915 | NRXN1 | 0.0338218 | -2.483810137 | |
| ENSG00000109472 | CPE | 0.0122881 | -2.483665122 | |
| ENSG00000182054 | IDH2 | 0.00830821 | -2.479731177 | |
| ENSG00000162482 | AKR7A3 | 0.00337364 | -2.478485294 | |
| ENSG00000139144 | PIK3C2G | 0.00337364 | -2.475850956 | |
| ENSG00000145284 | SCD5 | 0.0161348 | -2.459280274 | |
| ENSG00000188783 | PRELP | 0.00337364 | -2.440686536 | |
| ENSG00000170962 | PDGFD | 0.00337364 | -2.419301051 | |
| ENSG00000166123 | GPT2 | 0.0470172 | -2.405251797 | |
| ENSG00000169715 | MT1E | 0.00337364 | -2.403358975 | |
| ENSG00000090659 | CD209 | 0.0214509 | -2.395253268 | |
| ENSG00000163520 | FBLN2 | 0.0379226 | -2.373614178 | |
| ENSG00000233757 | [AC092835.2](http://asia.ensembl.org/Homo_sapiens/Gene/Summary?g=ENSG00000233757&db=core) | 0.0363824 | -2.371985435 | |
| ENSG00000078295 | ADCY2 | 0.031333 | -2.370034319 | |
| ENSG00000103942 | HOMER2 | 0.0178306 | -2.359260246 | |
| ENSG00000069702 | TGFBR3 | 0.00830821 | -2.357961382 | |
| ENSG00000169031 | COL4A3 | 0.00830821 | -2.353615139 | |
| ENSG00000184347 | SLIT3 | 0.0104168 | -2.352216812 | |
| ENSG00000157551 | KCNJ15 | 0.0104168 | -2.342406963 | |
| ENSG00000137070 | IL11RA | 0.0246715 | -2.320695561 | |
| ENSG00000156218 | ADAMTSL3 | 0.00337364 | -2.305579186 | |
| ENSG00000125148 | MT2A | 0.00830821 | -2.29929657 | |
| ENSG00000196569 | LAMA2 | 0.00337364 | -2.28111537 | |
| ENSG00000148357 | HMCN2 | 0.00830821 | -2.264092947 | |
| ENSG00000076864 | RAP1GAP | 0.00337364 | -2.261702909 | |
| ENSG00000107560 | RAB11FIP2 | 0.00337364 | -2.251830684 | |
| ENSG00000154736 | ADAMTS5 | 0.00830821 | -2.246500673 | |
| ENSG00000137819 | PAQR5 | 0.0198214 | -2.24257997 | |
| ENSG00000109819 | PPARGC1A | 0.00337364 | -2.236164488 | |
| ENSG00000189058 | APOD | 0.00598548 | -2.209270722 | |
| ENSG00000112936 | C7 | 0.00337364 | -2.191836251 | |
| ENSG00000165185 | KIAA1958 | 0.0104168 | -2.188237876 | |
| ENSG00000105426 | PTPRS | 0.00598548 | -2.169622936 | |
| ENSG00000083720 | OXCT1 | 0.00337364 | -2.165920804 | |
| ENSG00000073711 | PPP2R3A | 0.0178306 | -2.163337176 | |
| ENSG00000165914 | TTC7B | 0.031333 | -2.15466394 | |
| ENSG00000123243 | ITIH5 | 0.00598548 | -2.144298485 | |
| ENSG00000117322 | CR2 | 0.0233263 | -2.143510847 | |
| ENSG00000119938 | PPP1R3C | 0.0178306 | -2.138880783 | |
| ENSG00000169418 | NPR1 | 0.043051 | -2.135125294 | |
| ENSG00000211445 | GPX3 | 0.00337364 | -2.129387896 | |
| ENSG00000136872 | ALDOB | 0.0246715 | -2.12377435 | |
| ENSG00000100139 | MICALL1 | 0.0246715 | -2.118118221 | |
| ENSG00000102547 | CAB39L | 0.00337364 | -2.117961045 | |
| ENSG00000143382 | ADAMTSL4 | 0.0178306 | -2.111219001 | |
| ENSG00000003989 | SLC7A2 | 0.00598548 | -2.106945999 | |
| ENSG00000180251 | SLC9A4 | 0.0122881 | -2.105658539 | |
| ENSG00000154556 | SORBS2 | 0.00337364 | -2.10423144 | |
| ENSG00000066230 | SLC9A3 | 0.00337364 | -2.086950506 | |
| ENSG00000159224 | GIP | 0.00337364 | -2.08592088 | |
| ENSG00000184454 | NCMAP | 0.0298472 | -2.084786631 | |
| ENSG00000151376 | ME3 | 0.00337364 | -2.079384927 | |
| ENSG00000128573 | FOXP2 | 0.0280832 | -2.046233822 | |
| ENSG00000077044 | DGKD | 0.00337364 | -2.035084834 | |
| ENSG00000151892 | GFRA1 | 0.0122881 | -2.017068053 | |
| ENSG00000088280 | ASAP3 | 0.00337364 | -1.985634638 | |
| ENSG00000149451 | ADAM33 | 0.0142731 | -1.980182553 | |
| ENSG00000150893 | FREM2 | 0.0142731 | -1.969210962 | |
| ENSG00000164236 | ANKRD33B | 0.0142731 | -1.909037172 | |
| ENSG00000167757 | KLK11 | 0.0178306 | -1.905004974 | |
| ENSG00000164180 | TMEM161B | 0.00830821 | -1.904632216 | |
| ENSG00000139112 | GABARAPL1 | 0.00337364 | -1.901846121 | |
| ENSG00000196177 | ACADSB | 0.043051 | -1.896094971 | |
| ENSG00000115457 | IGFBP2 | 0.00337364 | -1.894562859 | |
| ENSG00000004799 | PDK4 | 0.00337364 | -1.882479323 | |
| ENSG00000148218 | ALAD | 0.00337364 | -1.879659503 | |
| ENSG00000198721 | ECI2 | 0.00598548 | -1.879077289 | |
| ENSG00000172164 | SNTB1 | 0.0178306 | -1.870005334 | |
| ENSG00000132359 | RAP1GAP2 | 0.00598548 | -1.868894322 | |
| ENSG00000140450 | ARRDC4 | 0.00337364 | -1.868877465 | |
| ENSG00000124406 | ATP8A1 | 0.0326031 | -1.830402223 | |
| ENSG00000022267 | FHL1 | 0.00337364 | -1.820723059 | |
| ENSG00000149294 | NCAM1 | 0.0418141 | -1.814229436 | |
| ENSG00000214944 | ARHGEF28 | 0.00598548 | -1.804127656 | |
| ENSG00000158966 | CACHD1 | 0.00337364 | -1.797952187 | |
| ENSG00000143365 | RORC | 0.00598548 | -1.793981295 | |
| ENSG00000140092 | FBLN5 | 0.0246715 | -1.791531482 | |
| ENSG00000157404 | KIT | 0.00598548 | -1.781327778 | |
| ENSG00000106804 | C5 | 0.0233263 | -1.772857178 | |
| ENSG00000180354 | MTURN | 0.0104168 | -1.769652461 | |
| ENSG00000142611 | PRDM16 | 0.0178306 | -1.767968761 | |
| ENSG00000137033 | IL33 | 0.0214509 | -1.75461792 | |
| ENSG00000198626 | RYR2 | 0.0161348 | -1.754019163 | |
| ENSG00000132405 | TBC1D14 | 0.0104168 | -1.745560123 | |
| ENSG00000185551 | NR2F2 | 0.00598548 | -1.731777647 | |
| ENSG00000198846 | TOX | 0.0349921 | -1.67059379 | |
| ENSG00000117266 | CDK18 | 0.00337364 | -1.66706402 | |
| ENSG00000183579 | ZNRF3 | 0.0418141 | -1.65952119 | |
| ENSG00000174136 | RGMB | 0.0142731 | -1.64735287 | |
| ENSG00000183762 | KREMEN1 | 0.0161348 | -1.642480017 | |
| ENSG00000250722 | SEPP1 | 0.00337364 | -1.632109798 | |
| ENSG00000107537 | PHYH | 0.0495902 | -1.625784845 | |
| ENSG00000170145 | SIK2 | 0.0338218 | -1.589619391 | |
| ENSG00000184012 | TMPRSS2 | 0.0246715 | -1.584206391 | |
| ENSG00000019102 | VSIG2 | 0.0161348 | -1.572568687 | |
| ENSG00000001561 | ENPP4 | 0.0246715 | -1.566709674 | |
| ENSG00000139044 | B4GALNT3 | 0.0326031 | -1.559773255 | |
| ENSG00000143409 | FAM63A | 0.0262905 | -1.559138986 | |
| ENSG00000162407 | PLPP3 | 0.00337364 | -1.553380774 | |
| ENSG00000082438 | COBLL1 | 0.0407413 | -1.544961223 | |
| ENSG00000100307 | CBX7 | 0.00337364 | -1.542071588 | |
| ENSG00000107331 | ABCA2 | 0.00598548 | -1.532869838 | |
| ENSG00000163050 | COQ8A | 0.0104168 | -1.52417047 | |
| ENSG00000075239 | ACAT1 | 0.0298472 | -1.495402096 | |
| ENSG00000074527 | NTN4 | 0.00337364 | -1.483474349 | |
| ENSG00000268043 | NBPF12 | 0.043051 | -1.443491413 | |
| ENSG00000122042 | UBL3 | 0.0142731 | -1.426474836 | |
| ENSG00000186480 | INSIG1 | 0.0443614 | -1.424631436 | |
| ENSG00000120053 | GOT1 | 0.0443614 | -1.420271998 | |
| ENSG00000105875 | WDR91 | 0.0326031 | -1.412704946 | |
| ENSG00000072422 | RHOBTB1 | 0.0161348 | -1.40342246 | |
| ENSG00000168497 | SDPR | 0.0326031 | -1.38524008 | |
| ENSG00000183087 | GAS6 | 0.0262905 | -1.364595639 | |
| ENSG00000133065 | SLC41A1 | 0.0418141 | -1.313400274 | |
| ENSG00000158828 | PINK1 | 0.0338218 | -1.313374542 | |
| ENSG00000198889 | DCAF12L1 | 0.00337364 | -0.474046599 | |

DEmRNA: differentially expressed mRNAs; STAD: stomach adenocarcinoma; FDR: false discovery rate; FC: fold change.
